# Chronic low-grade inflammation, genetic susceptibility, and the risk of multiple sclerosis: a prospective cohort study

**DOI:** 10.3389/fimmu.2026.1805464

**Published:** 2026-08-26

**Authors:** Yizhou Cai, Yujia Bao, Yongxuan Li, Jiawei Gu, Qingqing Ran, Yibin Zhou, Linjuan Dong, Dunjia Wang, Xia Zhao, Yuping Cheng, Dandan He

**Affiliations:** 1 Shanghai Minhang District Center for Disease Control and Prevention (Shanghai Minhang District Health Supervision Institute), Shanghai, China; 2 School of Public Health, Shanghai Jiao Tong University School of Medicine, Shanghai, Shanghai, China

**Keywords:** brain health, cohort study, epidemiology, genetic risk, low-grade inflammation, multiple sclerosis

## Abstract

**Background:**

Immune dysregulation is central to multiple sclerosis (MS) pathogenesis, yet prospective evidence linking peripheral low-grade inflammation (LGI) to MS risk remains limited and inconsistent. We investigated the association of LGI with incident MS risk and MS-related brain structural measures in a large prospective cohort.

**Methods:**

We analyzed data from 377,487 UK Biobank participants aged 40 to 69 years. A composite inflammation score (INFLA-score) was derived from C-reactive protein, white blood cell count, platelet count, and the neutrophil-to-lymphocyte ratio. Cox proportional hazards models assessed the association between LGI level and the incidence of multiple sclerosis (MS). Linear regression models evaluated the relationship between LGI level and the volume, cortical thickness, and surface area of six MS-related brain regions. Subgroup and interaction analyses were conducted.

**Results:**

Over a median follow-up of 13.8 years, 432 incident MS cases were documented, with a higher incidence density in women than in men (10.8 per 100,000 person-years vs 5.8 per 100,000 person-years). High-level LGI was associated with an increased risk of MS (HR = 1.38; 95% confidence interval [CI]: 1.10–1.72). This association was generally consistent across sex and polygenic risk score categories (all *P* for interaction > 0.05), but was stronger among individuals with higher socioeconomic deprivation (HR = 1.73; 95% CI: 1.27–2.35; *P* for interaction = 0.035) and those with hypertension (HR = 2.67; 95% CI: 1.63–4.35; *P* for interaction = 0.002). High-level LGI was negatively correlated with the volumes of the thalamus, superior temporal gyrus, lateral orbitofrontal cortex, putamen, caudate nucleus, and supramarginal gyrus (all P < 0.05). The association between LGI and lower brain volumes was generally more pronounced in males, while no significant interaction was observed between LGI and genetic risk on brain morphometry (all P for interaction > 0.05).

**Conclusions:**

LGI is associated with an increased risk of MS and with adverse variations in brain morphometry in MS-related brain regions. These findings suggest that peripheral inflammation may serve as a potential marker for identifying individuals at elevated MS risk, warranting further investigation in interventional studies.

## 1 Introduction

Multiple sclerosis (MS) is a chronic inflammatory autoimmune disease that traditionally affects young adults aged 20 to 40, with females accounting for nearly three-quarters of cases (1, 2). However, recent evidence points to a growing prevalence of MS among older populations in developed regions, a trend likely attributable to demographic aging, advances in diagnostic capabilities, and the introduction of high-efficacy disease-modifying therapies that improve disease stability and extend patient longevity (3). The majority of patients initially present with a relapsing-remitting course (RRMS); however, as disease duration increases, incomplete recovery from relapses leads to cumulative disability and eventual transition to secondary progressive MS (SPMS) (3). This progressive decline is compounded by the accumulation of age-related comorbidities, which further exacerbate motor, cognitive, and psychiatric impairment (2, 4, 5). Given the expanding burden of MS in aging populations, identifying modifiable risk factors relevant to this demographic is of considerable clinical and public health importance.

Current evidence suggests that MS is a multifactorial disease influenced by genetic predisposition and environmental factors (1). Tissue damage in MS results from a complex and dynamic interplay involving the immune system, glia, and neurons (1). Findings from the International Multiple Sclerosis Genetics Consortium (IMSGC) have demonstrated that the majority of genetic risk loci for MS implicate immune-pathway genes, underscoring the central role of immune dysregulation in disease pathogenesis (6). Critically, the aging immune system is characterized by two interrelated phenomena—immunosenescence and chronic low-grade inflammation (LGI)—both of which may act upon this genetic substrate to alter MS risk (7) (8). Beyond biological aging, various contemporary lifestyle factors—including obesity, microbiome dysbiosis, dietary changes, and environmental toxin exposure—further promote a state of systemic LGI (9).

The LGI state, as an overproduction of peripheral pro-inflammatory mediators in the absence of overt clinical symptoms, has garnered growing interest in the neurology field (10–13). Gialluisi et al. demonstrated that a state of systemic LGI had a significant effect on symptoms and mortality in major depression (14). A meta-analysis revealed that elevated interleukin-6 (IL-6) and C-reactive protein (CRP) levels were associated with increased susceptibility to all-cause dementia (15), and systemic LGI has been linked to functional impairment in patients with Alzheimer’s disease (16). With respect to MS specifically, recent studies have reported a correlation between elevated neutrophil-to-lymphocyte ratio (NLR) and both the onset and progression of MS (17, 18). However, other studies have yielded inconsistent results (19, 20). These discrepancies may arise from ethnic or demographic differences, residual confounding, or methodological limitations—most existing studies are constrained by small sample sizes, retrospective designs, and assessment of inflammatory markers only at the time of diagnosis or hospital admission (21).

From a mechanistic standpoint, peripheral inflammatory cytokines can cross the blood–brain barrier (BBB), activate microglia and astrocytes, and disrupt synaptic plasticity and neuronal integrity (22). Consistent with this pathway, elevated inflammatory markers have been associated with reduced subcortical and cortical volumes in population-based studies (23, 24), and neuroimaging research in MS patients demonstrates widespread brain atrophy—particularly affecting the thalamus, caudate nucleus, striatum, and cerebellar cortex (25–27). These converging lines of evidence suggest that LGI may contribute to MS-related structural brain changes, yet this hypothesis remains to be fully examined in large population-based cohorts.

Building upon these considerations, we leveraged the UK Biobank resource to conduct a prospective cohort study evaluating the association between LGI and incident MS risk. LGI level was quantified using a composite inflammatory index, the INFLA-score, alongside its individual biomarkers. We further assessed whether genetic susceptibility potentially modulates this relationship and investigated the links between LGI and MS-related brain structural measures. These findings are intended to provide longitudinal epidemiological evidence that may facilitate risk stratification and inform the potential development of targeted intervention strategies for MS, particularly within the context of an aging population.

## 2 Methods

### 2.1 Study design and participants

The UK Biobank is a large cohort study that recruited about 500,000 individuals aged 40 to 69 in England, Scotland, and Wales. Baseline data were collected at 22 centers using touchscreen questionnaires, interviews, physical measurements, and genetic assessments, with informed consent, from 2006 to 2010. A linked electronic healthcare record is available for most participants, allowing researchers to analyze data from various sources. The follow-up period extended from the date of the baseline assessment until the earliest date of MS diagnosis, death, or the end of follow-up (December 19, 2022), whichever transpired first. This study was conducted under approval from the North West Multi-center Research Ethics Committee as a Research Tissue Bank approval. (ref. 21/NW/0157). Our study was conducted under Application Number 99001 (Approval date: 2023-01–31 to 2026-01-31).

In our study, we screened 502,369 participants from the UK Biobank. Participants were excluded if they met the following criteria: 1) pre-existing MS at baseline, identified via self-report or hospital inpatient records; 2) residence in rural areas — an exclusion necessitated to minimize potential confounding from urban-rural lifestyle variations highly correlated with LGI and ensure the completeness of medical records; 3) missing data on plasma samples, important covariates, or follow-up; 4) failure to meet genetic quality control standards, including a genotyping call rate <95%, excessive heterozygosity, or a mismatch between genetic and reported sex. Finally, 377,487 participants were included in the main analysis. Furthermore, a total of 32,117 participants were involved in the brain imaging analysis after excluding those with missing or abnormal MRI data.

### 2.2 Low-grade inflammation score

We computed the INFLA-score as an aggregated measure of LGI. The INFLA-score, containing CRP, white blood cell (WBC), platelets (PLT), and NLR, has been reported to be associated with diet and mental health in previous prospective studies (28, 29) (**Supplementary Figure 1**). To compute the INFLA-score, for all four components, laying in the highest deciles (7th to 10th) were assigned values from +1 to +4; while biomarker levels laying in the lowest deciles (1st to 4th) were given values from -4 to -1. The INFLA-score, ranging from -16 to +16, is the sum of these scores, with higher values indicating greater LGI (30). Scores are categorized into low-level and high-level based on the median INFLA-score. More details are displayed in **Supplementary Methods** and **Supplementary Table 1**.

### 2.3 Definitions of outcomes

The primary outcome in the study is the incidence of MS. We derived MS disease status from the UK Biobank “summary diagnoses” fields (UKB category 2002), which contains summary fields relating to diagnoses made during hospital inpatient admissions. Most data-fields in this category have a corresponding field showing the date each diagnosis code was first recorded in the inpatient data. MS cases were defined as participants with the International Classification of Diseases (ICD) (ICD-9: 340 & ICD-10: G35).

The secondary outcome of the study is the volumes, thickness and area of key brain structures. Brain imaging acquisition commenced in 2014, neuroimaging data were collected on a standard Siemens Skyra 3T scanner with a 32-channel head coil. Quality-controlled T1-weighted neuroimaging data were processed with FreeSurfer, surface templates were utilized to extract imaging derived phenotypes referring to atlas regions’ surface volume. According to previous studies based on MS, we selected six brain regions, including caudate nucleus, putamen, thalamus, lateral orbitofrontal cortex, supramarginal gyrus, and superior temporal gyrus. Meanwhile we calculated mean thickness and area of three cortical structures, including lateral orbitofrontal cortex, supramarginal gyrus, and superior temporal gyrus. MRI variables were adjusted for skull size and converted to Z scores for interpretation and comparison. The imaging data used in this study were collected between 2014 and 2020. More details of the above brain imaging phenotypes are displayed in **Supplementary Methods** and **Supplementary Table 2**.

### 2.4 Polygenic risk score

The procedure for genotyping and imputation in the UK Biobank has been described in detail previously(31). To construct the PRS, we used 110 single nucleotide polymorphisms (SNPs) reported by the IMSGC (32). However, due to restricted access to individual-level genotyping data, genotyping array and genetic ancestry principal components were not included as covariates in the PRS model. According to the number of risk alleles, each SNP was recorded as 0, 1 or 2 according to the number of risk estimate (β coefficient) on MS. The PRS was calculated using the equation:


$$\text{PRS}=\sum_{j=1}^{M}\beta_{j}\times {\text{SNP}}_{j}$$

, where M is the total number of SNPs, and β_j_ is per allele log odds ratio (OR) for MS associated with SNPj. Then participants were categorized into three groups: low (lowest tertile), intermediate (intermediate tertile), or high (highest tertile) genetic risk. The details of the SNPs and calculation of PRS are displayed in **Supplementary Table 3**.

### 2.5 Covariates

Relevant covariates were measured at baseline. Demographic variables included age, sex, index of multiple deprivation (IMD) scores, waist-to-hip ratio (WHR). Healthy lifestyle included never smoking (yes vs no), never drinking (yes vs no), regular physical activity, healthy sleep, and healthy diet. Regular physical activity was defined as at least 150 minutes of moderate activity per week or 75 minutes of vigorous activity per week (33). Sleep healthy scores were used to assess sleep behaviors (34), which included 5 aspects: chronotype, sleep duration, insomnia, snoring, and excessive daytime sleepiness. Each sleep factor was coded 1 if meeting the healthy criterion and 0 if not. We categorized it into “healthy sleep pattern” when the score ≥4. Healthy diet was based on consumption of at least 4 of 7 commonly eaten food groups, in accordance with dietary guidelines designed to enhance cardiometabolic health (33). Chronic disease conditions included hypertension, diabetes, and stroke. More details of variables are shown in **Supplementary Table 4**.

### 2.6 Statistical analysis

For differences in baseline characteristics across INFLA-score categories (low and high), we used the chi-squared (χ2) test for the variables. Baseline characteristics of these subsets are presented as the number (percentage).

The incidence density of MS was calculated for the overall cohort, as well as separately for women and men, and across three genetic risk groups (low, moderate, high). We used a Cox proportional hazards model to quantify associations of INFLA-score and its components (CRP, WBC, PLT, NLR) with incidence risk of MS with years of follow-up as the time metric, reporting hazard ratios (HR) and 95% CIs. Schoenfeld residuals were used to test the proportional hazard assumption. Three sets of models were applied. Model 1 did not adjust for any variables. Model 2 adjusted for age, sex, and IMD scores. Model 3 further adjusted for WHR, healthy lifestyle, and chronic disease conditions. Based on the model with full adjustments for potential confounders, subgroup analyses were further conducted by sex (male and female), PRS category (low, moderate, high), age (<65 years and ≥65 years), IMD scores (low and high), regular physical activity (yes and no), healthy sleep pattern (yes and no), hypertension (yes and no) and diabetes (yes and no). The likelihood ratio test between models with and without the interaction term was conducted to estimate the significance of the interaction effects.

Additionally, we used multiple linear regression analyses to estimate the associations of LGI and the inflammation biomarkers with brain structural traits related to MS (including volume of caudate nucleus, putamen, thalamus, lateral orbitofrontal cortex, supramarginal gyrus and superior temporal gyrus, area of lateral orbitofrontal cortex, supramarginal gyrus and superior temporal gyrus, and mean thickness of lateral orbitofrontal cortex, supramarginal gyrus and superior temporal gyrus) in the model with full adjustments (including age, sex, IMD scores, WHR, no smoking, no drinking, regular physical activity, healthy sleep pattern, healthy diet, and chronic health conditions such as hypertension, diabetes, and stroke). The estimates are shown as *β*s and 95% CIs. As all brain structural measures were standardized via Z-transformation, these *β* coefficients represent the mean difference in standard deviation (SD) units for participants in the high-level group compared with the low-level reference group. Multicollinearity assumptions were tested by the variance inflation factor (VIF), and the value of all variables was below five. Subgroup analyses were also conducted for brain structural traits. Interaction significance was determined via likelihood ratio tests, with nominal and false-discovery rate (FDR) -corrected P values reported to distinguish robust associations from potential multiple testing artifacts.

Furthermore, we performed a series of sensitivity analyses: 1) We exclude the non-White participants from the cohort, as the non-White participants only constitute approximately 3% of the dataset. 2) We observed the association of the COVID-19 pandemic with mortality (35, 36), and moved the follow-up deadline forward to December 31, 2019. 3) We changed the covariate of never drinking (yes vs no) to moderate drinking (yes vs no), and moderate drinking was defined as the average daily intake ≤16g of pure alcohol (2 units of alcohol) for both men and women. 4) To address potential delayed case ascertainment or reverse causation, we conducted lag analyses excluding participants diagnosed with MS within the first two or five years of follow-up.

All statistical analyses were performed with R software, version 4.2.1, we used package ‘survival’ for Cox proportional hazards regression, ‘stats’ for logistic regression and other statistical analyses, and ‘forestmodel’ for the creation of forest plots. Two-sided p <0.05 was considered statistically significant.

## 3 Result

### 3.1 Social demographic characteristics of participants

**Figure 1:**
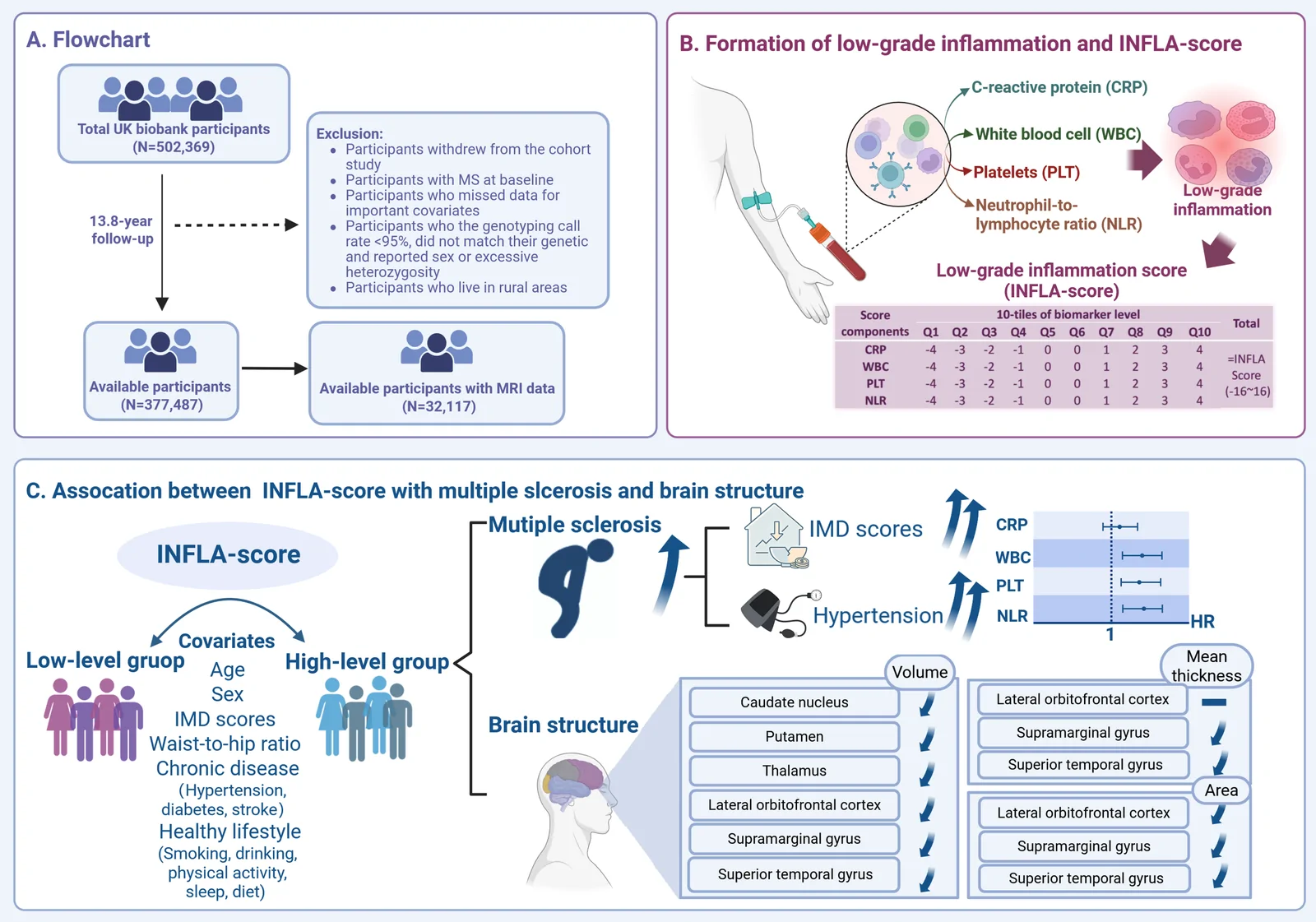
Study flowchart. **(A)** Population flowchart. **(B)** The calculation of immune biomarkers and an aggregated inflammation score (INFLA-score). **(C)** The main results of the study. IMD, Index of Multiple Deprivation.

A total of 377,487 participants were included in this study, with a median follow-up of 13.8 years, amounting to 5,081,861 total person-years of observation (**Figure 1**). During the follow-up period, 432 participants developed MS, with a mean age at diagnosis of 61.4 (± 8.8) years. The overall incidence rate was 8.5 per 100,000 person-years; the sex-specific rates were 5.8 per 100,000 person-years for males and 10.8 per 100,000 person-years for females (**Supplementary Table 5**). **Table 1** presents the baseline characteristics of the participants in this study by LGI. We found that participants with the higher level of LGI tended to be more likely observed among people aged more than 65 years, females, and those with high index of IMD scores, poor WHR. Participants with low-level LGI were more likely to have regular physical activity, healthy sleep, and healthy diet.

**Table 1:** Population characteristics included in the study.

Characteristic	All samples (N = 377487)	Low-grade inflammation^a^		
		Low-level (N = 200578)	High-level (N = 176909)	*P* value
Age				<0.001
<65	306637 (81.2%)	165723 (82.6%)	140914 (79.7%)	
≥65	70850 (18.8%)	34855 (17.4%)	35995 (20.3%)	
Sex				<0.001
Female	203980 (54.0%)	103660 (51.7%)	100320 (56.7%)	
IMD scores^b^				<0.001
Low	188948 (50.1%)	105375 (52.5%)	83573 (47.2%)	
WHR^c^				0.001
Ideal	191019 (50.7%)	112526 (56.2%)	78493 (44.5%)	
Lifestyle				
Never smoking	205645 (54.8%)	115642 (57.9%)	90003 (51.2%)	<0.001
Never drinking	30012 (8.0%)	14673 (7.3%)	15339 (8.7%)	<0.001
Regular physical activity^d^	165827 (54.4%)	94382 (57.1%)	71445 (51.2%)	<0.001
Healthy sleep pattern^e^	199333 (52.8%)	110332 (55.0%)	89001 (50.3%)	<0.001
Healthy diet^f^	143838 (38.1%)	83233 (41.5%)	60605 (34.3%)	<0.001
Chronic disease condition				
Hypertension	108411 (28.7%)	49300 (24.6%)	59111 (33.4%)	<0.001
Diabetes	19789 (5.2%)	8513 (4.2%)	11276 (6.4%)	<0.001
Stroke	6090 (1.6%)	2598 (1.3%)	3492 (2.0%)	<0.001
Cases of MS	432 (0.11%)	184 (0.09%)	248 (0.14%)	<0.001

IMD scores, index of multiple deprivation scores; WHR, Waist-to-hip ratio; MS, multiple sclerosis. ^a^Low-grade inflammation was categorized into low-level and high-level according to the median of INFLA-score; ^b^IMD scores were categorized into low and high levels by the median. ^c^Waist-to-hip ratio was classified into ideal and poor categories with 0.85 for women and 0.90 for men as the cutoff value. ^d^Healthy sleep pattern was defined as healthy sleep score ≥4, and healthy sleep score include 5 aspects: chronotype, sleep duration, insomnia, snoring, and excessive daytime sleepiness, healthy sleep factors were defined as early chronotype (“morning” or “morning than evening”); sleep 7–8 h/day; reported never/rarely or sometimes insomnia symptoms; no self-reported snoring; and no excessive daytime sleepiness (“never/rarely” or “sometimes”). Each sleep factor was coded 1 if meeting the healthy criterion and 0 if not. ^e^Regular physical activity was defined as meeting the American Heart Association recommendations of at least 150 minutes of moderate activity per week or 75 minutes of vigorous activity per week (or an equivalent combination) or engaging in moderate physical activity at least 5 days a week or vigorous activity once a week. ^f^Healthy diet was based on consumption of at least 4 of 7 commonly eaten food groups following recommendations on dietary priorities for cardiometabolic health.

### 3.2 Association between LGI and the incidence risk of MS

**Figure 2:**
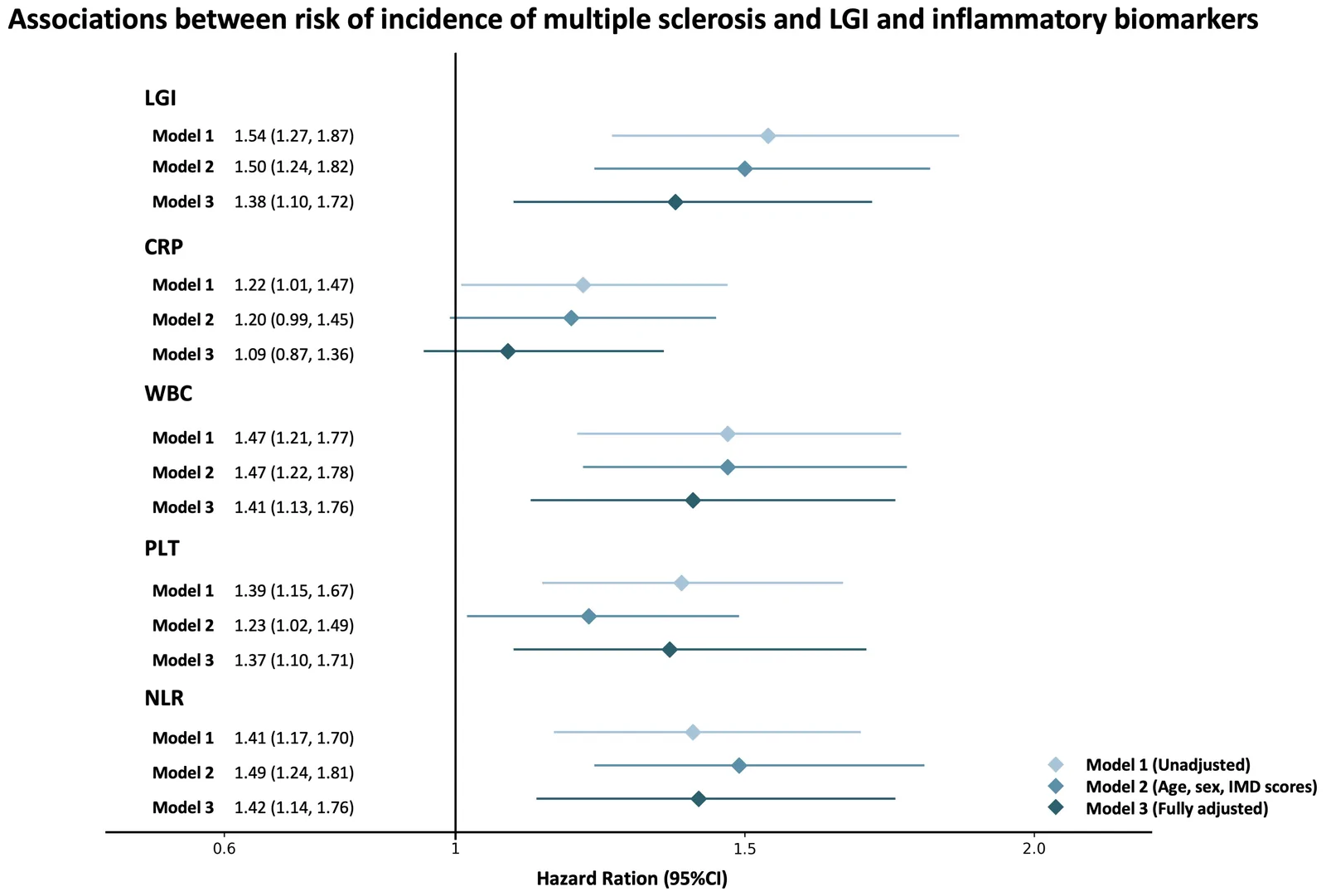
Forest plot of the associations between low-grade inflammation and inflammation biomarkers with the incidence risk of multiple sclerosis. Hazard ratios (HRs) and 95% confidence intervals (CIs) were estimated using Cox proportional hazards regression models, with the low-level group as reference. Dots with error bars colored in light to deep blue were hazard ratios with 95% CIs for model 1, model 2 and model 3, respectively. The vertical dashed line indicates the null value (HR = 1.00). Model 1 did not adjust for any variables. Model 2 adjusted for age, sex, and index of multiple deprivation (IMD) scores. Model 3 adjusted for age, sex, IMD scores, waist-to-hip ratio, no smoking, no drinking, regular physical activity, healthy sleep pattern, healthy diet, and chronic disease conditions including hypertension, diabetes, and stroke. LGI, low-grade inflammation; CRP, C-reactive protein; WBC, white blood cell count; PLT, platelet count; NLR, neutrophil-to-lymphocyte ratio; IMD, Index of Multiple Deprivation; CI, confidence interval.

The association between LGI and inflammation markers with MS occurrence is shown in **Figure 2**, **Supplementary Tables 6**, **7**. Sensitivity analyses confirmed the robustness of our main results (**Supplementary Table 8**). When adjusting for multiple factors using proportional Cox regression models (model 3), the LGI was significantly associated with an increased incidence risk of MS (HR = 1.38, 95% CI: 1.10–1.72). Among the individual inflammation biomarkers, NLR (HR = 1.42, 95% CI: 1.14–1.76), WBC (HR = 1.41, 95% CI: 1.13–1.76), and PLT (HR = 1.37, 95% CI: 1.10–1.71) remained significantly associated with MS risk after adjusting for all covariates. However, the association between CRP and MS incidence was attenuated and no longer statistically significant in the fully adjusted model (HR = 1.09, 95% CI: 0.87–1.36).

Subgroup analyses were conducted to examine potential effect modification of the above associations (**Table 2**; **Supplementary Table 9**). No significant interactions were observed between any inflammation biomarker and either sex, PRS category, healthy sleep patterns or regular physical activity (all *P* for interaction > 0.05), indicating that the associations were generally consistent across these subgroups. In contrast, significant effect modification was detected by age (<65 vs. ≥65 years), socioeconomic deprivation, hypertension, and diabetes status. When stratified by age (<65 vs. ≥65 years), a significant interaction was detected only for PLT (*P* for interaction = 0.047), with the association being significant in participants aged <65 years (HR = 1.49, 95% CI: 1.18–1.88) but not in those aged ≥65 years (HR = 0.72, 95% CI: 0.37–1.42). For IMD scores, significant interactions were found for LGI (P for interaction = 0.035) and WBC (*P* for interaction = 0.048). The association between elevated LGI and incident MS was stronger among individuals with higher deprivation (HR = 1.73, 95% CI: 1.27–2.35) than among those with lower deprivation (HR = 1.07, 95% CI: 0.78–1.48). Similarly, the WBC–MS association was more pronounced in the high IMD group (HR = 1.75, 95% CI: 1.30–2.37) compared to the low IMD group (HR = 1.10, 95% CI: 0.80–1.52). For hypertension, significant interactions were observed for LGI (*P* for interaction = 0.002) and CRP (*P* for interaction = 0.005). The LGI–MS association was markedly stronger among participants with hypertension (HR = 2.67, 95% CI: 1.63–4.35) compared to those without (HR = 1.12, 95% CI: 0.87–1.44). Likewise, elevated CRP was significantly associated with MS risk only in the hypertension subgroup (HR = 1.83, 95% CI: 1.19–2.82) but not in those without hypertension (HR = 0.89, 95% CI: 0.68–1.16). For diabetes, a significant interaction was detected for CRP (*P* for interaction = 0.010). The CRP–MS association was stronger among participants with diabetes (HR = 1.81, 95% CI: 1.16–2.82) than among those without (HR = 0.90, 95% CI: 0.66–1.22).

**Table 2:** Associations of low-grade inflammation and the inflammation biomarkers with incidence risks of multiple sclerosis across subgroups.

Subgroup	LGI^a^		CRP^b^		WBC^c^		PLT^d^		NLR^e^	
	HR (95% CI)	*P* for interaction	HR (95% CI)	*P* for interaction	HR (95% CI)	*P* for interaction	HR (95% CI)	*P* for interaction	HR (95% CI)	*P* for interaction
Sex										
Male	1.35 (0.93, 1.96)	0.911	1.13 (0.77, 1.65)	0.803	1.76 (1.21, 2.57)	0.153	1.49 (1.02, 2.17)	0.608	1.43 (0.99, 2.07)	0.971
Female	1.39 (1.06, 1.82)		1.07 (0.81, 1.40)		1.26 (0.97, 1.65)		1.32 (1.01, 1.72)		1.42 (1.09, 1.85)	
PRS category										
Low	1.09 (0.70, 1.72)	0.468	0.80 (0.50, 1.29)	0.107	1.46 (0.93, 2.29)	0.42	1.42 (0.90, 2.23)	0.537	1.03 (0.65, 1.63)	0.281
Moderate	1.38 (0.96, 2.01)		0.96 (0.66, 1.40)		1.17 (0.80, 1.69)		1.59 (1.10, 2.29)		1.50 (1.04, 2.16)	
High	1.56 (1.11, 2.18)		1.41 (1.01, 1.98)		1.62 (1.16, 2.26)		1.20 (0.86, 1.68)		1.62 (1.16, 2.25)	
Age										
<65 years	1.43 (1.13, 1.81)	0.362	1.08 (0.85, 1.38)	0.903	1.47 (1.16, 1.86)	0.319	1.49 (1.18, 1.88)	0.047	1.39 (1.11, 1.75)	0.619
≥65 years	1.05 (0.56, 1.95)		1.13 (0.61, 2.10)		1.05 (0.56, 1.96)		0.72 (0.37, 1.42)		1.65 (0.88, 3.08)	
IMD scores^f^										
Low	1.07 (0.78, 1.48)	0.035	0.95 (0.68, 1.32)	0.262	1.10 (0.80, 1.52))	0.048	1.40 (1.02, 1.92)	0.856	1.29 (0.94, 1.77)	0.403
High	1.73 (1.27, 2.35)		1.22 (0.90, 1.65)		1.75 (1.30, 2.37)		1.35 (1.00, 1.81)		1.55 (1.15, 2.08)	
Hypertension										
Yes	2.67 (1.63, 4.35)	0.002	1.83 (1.19, 2.82)	0.005	1.80 (1.18, 2.76)	0.187	1.74 (1.15, 2.64)	0.186	1.37 (0.91, 2.07)	0.838
No	1.12 (0.87, 1.44)		0.89 (0.68, 1.16)		1.29 (1.00, 1.67)		1.25 (0.97, 1.62)		1.44 (1.12, 1.86)	
Diabetes										
Yes	1.88 (1.21, 2.92)	0.132	1.81 (1.16, 2.82)	0.01	1.58 (1.07, 2.32)	0.569	1.83 (1.25, 2.68)	0.074	1.37 (0.94, 1.99)	0.808
No	1.27 (0.97, 1.66)		0.90 (0.66, 1.22)		1.38 (1.05, 1.80)		1.20 (0.92, 1.57)		1.45 (1.11, 1.89)	

LGI, low-grade inflammation; HR, Hazard ratios; CI, confidence intervals; CRP, C-reactive protein; WBC, white blood cell; PLT, platelets; NLR, neutrophil to lymphocyte ratio; PRS, polygenic risk scores; IMD scores, index of multiple deprivation scores.

a The reference is low-level LGI; ^b^The reference is low-level CRP; ^c^The reference is low-level WBC; ^d^The reference is low-level PLT; ^e^The reference is low-level NLR; ^f^IMD scores were categorized into low and high levels by the median.

### 3.3 Association between LGI and brain structures

**Figure 3:**
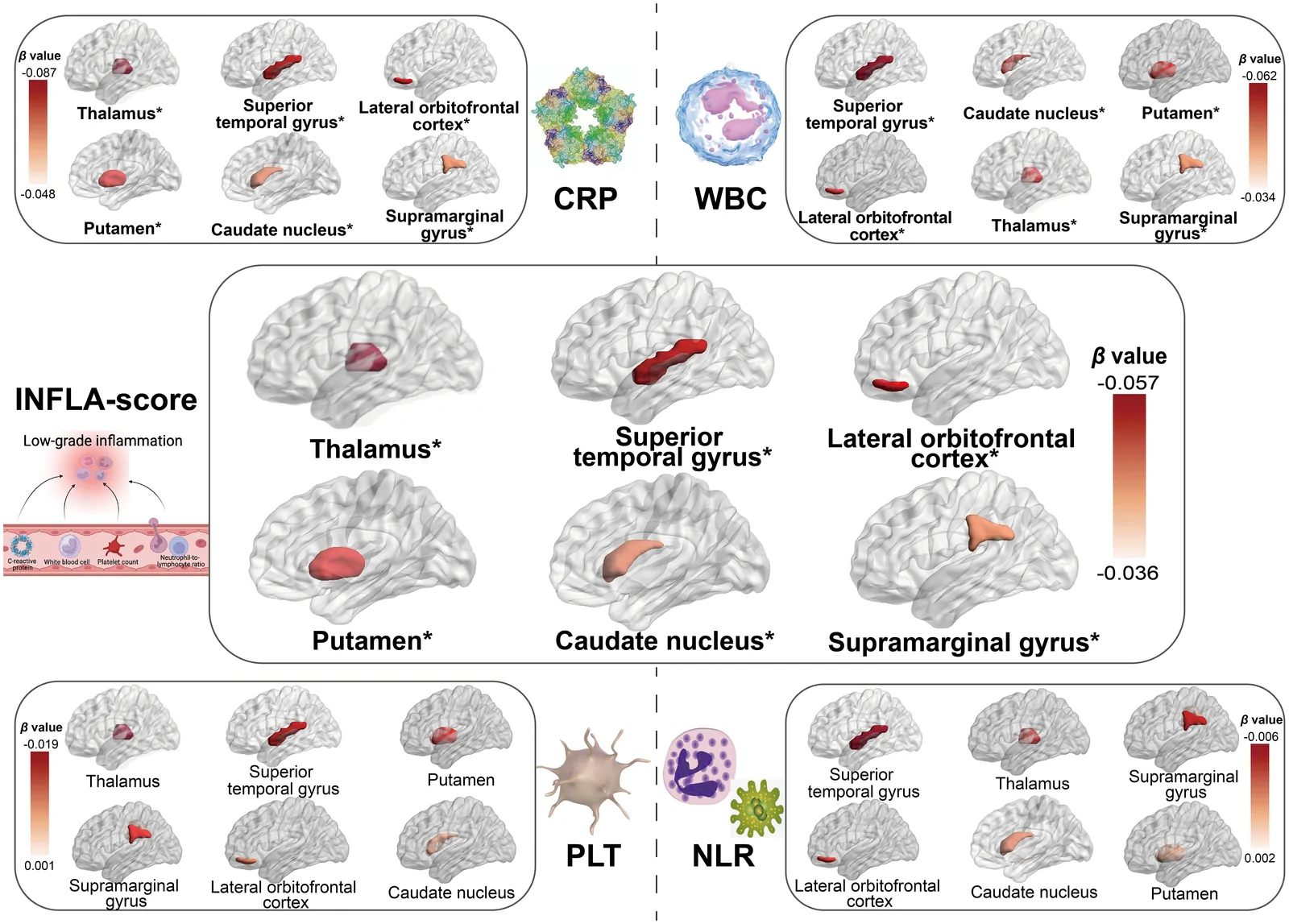
Associations of low-grade inflammation and the inflammation biomarkers with the volumes of brain structures. Multiple linear regression analyses were employed to estimate the associations of low-grade inflammation (LGI) and the inflammation biomarkers with volume of six brain structures related to multiple sclerosis. The inflammation biomarkers included C-reactive protein (CRP), white blood cell count (WBC), platelets (PLT) and the neutrophil to lymphocyte ratio (NLR). The model was adjusted for age, sex, index of multiple deprivation scores, waist-to-hip ratio, no smoking, no drinking, regular physical activity, healthy sleep pattern, healthy diet, and chronic health conditions such as hypertension, diabetes, and stroke. Data were presented as the *β*s. *p value FDR < 0.05. CRP, C-reactive protein; WBC, white blood cell count; PLT, platelet count; NLR, neutrophil-to-lymphocyte ratio.

**Figure 4:**
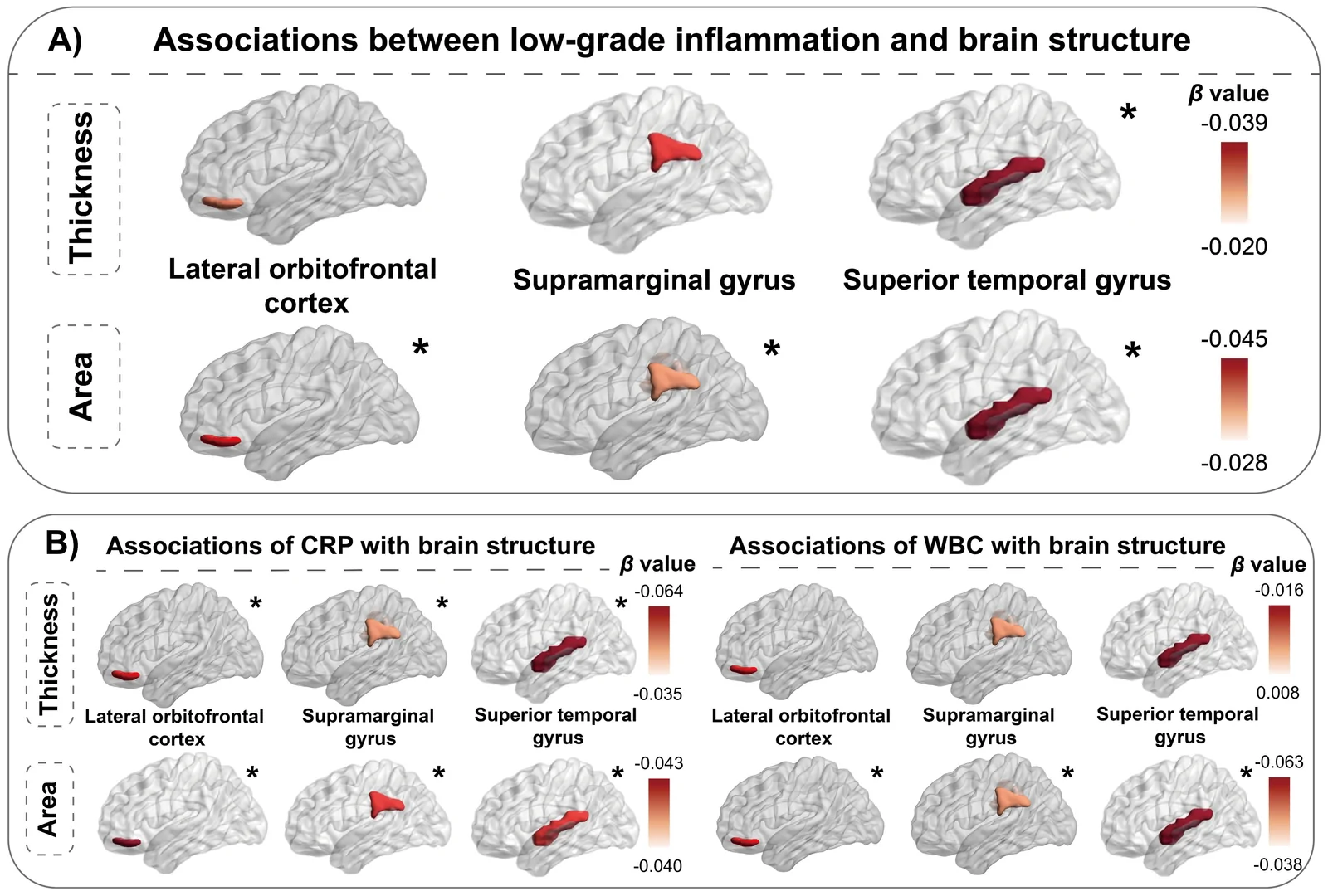
Associations of low-grade inflammation, CRP, and WBC with the thickness and area of brain structures. **(A)** Associations between low-grade inflammation and brain structure. **(B)** Associations of CRP and WBC with brain structure. Multiple linear regression analyses were utilized to estimate the associations of low-grade inflammation (LGI), C-reactive protein (CRP) and white blood cell count (WBC) with the thickness and area of six brain structures related to multiple sclerosis. The model was adjusted for age, sex, index of multiple deprivation scores, waist-to-hip ratio, no smoking, no drinking, regular physical activity, healthy sleep pattern, healthy diet, and chronic health conditions such as hypertension, diabetes, and stroke. Data were presented as the bs. *p value FDR < 0.05. CRP, C-reactive protein; WBC, white blood cell count.

In fully adjusted models with FDR correction, LGI was negatively associated with the volumes of all six MS-related brain regions identified *a priori* from the literature (**Figure 3**; **Supplementary Table 10**), including thalamus (*β:* -0.057, 95% CI: -0.079, -0.035); superior temporal gyrus (*β*: -0.053, 95% CI: -0.076, -0.031); lateral orbitofrontal cortex (*β*: -0.050, 95% CI: -0.072, -0.027); putamen (*β*: -0.047, 95% CI: -0.069, -0.025); caudate nucleus (*β*: -0.041, 95% CI: -0.064, -0.017); supramarginal gyrus (*β*: -0.036, 95% CI: -0.058, -0.013). Higher LGI levels were also associated with lower mean thickness of the superior temporal gyrus (*β*: -0.039, 95% CI: -0.062, -0.015) and smaller surface areas of the superior temporal gyrus (*β*: -0.045, 95% CI: -0.067, -0.023), lateral orbitofrontal cortex (*β*: -0.042, 95% CI: -0.064, -0.020) and supramarginal gyrus (*β*: -0.028, 95% CI: -0.050, -0.006). Consistent with the associations observed for the composite LGI score, higher CRP and WBC were associated with lower volumes across these imaging phenotypes. Notably, while CRP demonstrated inverse associations with both cortical thickness and surface area, WBC counts showed significant relationships exclusively with the surface areas of the three cortical structures (**Figure 4**; **Supplementary Table 10**).

Subgroup analyses indicated that the negative associations between LGI and regional brain volumes were more pronounced in males than in females, with significant interactions observed for the putamen (male: *β*: -0.077, 95% CI:-0.109, -0.046; female: *β*: -0.020,95% CI: -0.050, 0.009, *P* for interaction = 0.036), thalamus (male: *β*: -0.088, 95% CI:-0.120, -0.057; female: *β*: -0.030,95% CI: -0.060, 0.000, *P* for interaction = 0.034), lateral orbitofrontal cortex (male: *β*: -0.080, 95% CI:-0.112, -0.048; female: *β*: -0.023,95% CI: -0.053, 0.008, *P* for interaction = 0.038), and superior temporal gyrus (male: *β*: -0.087, 95% CI:-0.119, -0.054; female: *β*: -0.024,95% CI: -0.054, 0.007, *P* for interaction = 0.030) (**Supplementary Table 11**). Similarly, the negative correlation between LGI and the mean cortical thickness of the superior temporal gyrus (male: *β*: -0.073, 95% CI: -0.107, -0.039; female: *β*: -0.008,95% CI: --0.040, 0.024, *P* for interaction = 0.030) and supramarginal gyrus (male: *β*: -0.067, 95% CI: -0.102, -0.033; female: *β*: 0.015,95% CI: -0.018, 0.047, *P* for interaction = 0.008) were stronger in males participants than in females participants. No significant sex-based interactions were observed for the associations of CRP or PLT with brain structural measures (all *P* for interaction > 0.05). Meanwhile, the associations of NLR with brain volume, mean thickness, and surface area were in opposite directions between males and females—inverse in males and positive in females—except for caudate nucleus volume and supramarginal gyrus surface area (**Supplementary Tables 12, 13**).

In genetic risk-stratified analyses, we observed consistent negative associations between high-level LGI and subcortical volumes across strata, particularly in the thalamus (low genetic risk: *β*:−0.050, 95% CI: −0.088, −0.012; moderate: *β*: −0.062, 95% CI: −0.100, −0.025; high: *β*: −0.060, 95% CI:−0.098, −0.022), caudate nucleus (moderate: *β*: −0.052, 95% CI: −0.092, −0.012; high: *β*: −0.059, 95% CI: −0.099, −0.019), and putamen (low: *β*: −0.050, 95% CI: −0.088, −0.013; moderate: *β*: −0.057, 95% CI: −0.094, −0.020). For cortical volumes, high-level LGI was consistently associated with the superior temporal gyrus (moderate: *β*: −0.058, 95% CI: −0.096, −0.020; high: *β*: −0.067, 95% CI: −0.105, −0.029), lateral orbitofrontal cortex (moderate: *β*: −0.051, 95% CI: −0.089, −0.013; high: *β*: −0.058, 95% CI: −0.096, −0.020), and supramarginal gyrus (high: *β*: −0.051, 95% CI: −0.089, −0.013). Regarding cortical surface area, significant negative associations were observed only in the high genetic risk group for the lateral orbitofrontal cortex (*β*: −0.054, 95% CI: −0.092, −0.017) and superior temporal gyrus (*β*: −0.058, 95% CI: −0.096, −0.021). No significant associations were detected for cortical thickness in any subgroup. Despite the pattern of more pronounced associations among individuals with higher genetic risk, no statistically significant interactions between LGI and genetic risk strata were detected for any brain structural measure (all *P f*or interaction > 0.05, **Supplementary Table 14**).

## 4 Discussion

In this large prospective cohort study, we found that high-level LGI was associated with an increased risk of incident MS, and comparable associations were observed for PLT, WBC, and NLR individually. This association was more pronounced among females and among individuals with high genetic risk, greater socioeconomic deprivation, or comorbid hypertension. Furthermore, cross-sectional neuroimaging analyses revealed significant inverse associations between systemic LGI and the volumes, mean thickness, and surface area of six MS-related brain regions. These associations were notably more pronounced among male participants.

Our finding that high-level LGI was associated with an increased risk of incident MS is consistent with prior evidence linking peripheral inflammatory markers to a range of neurological and psychiatric conditions, including dementia (15), Alzheimer’s disease (16), and major depression (14, 37). Whereas most previous studies relied on single biomarkers assessed cross-sectionally, the present study employed a composite inflammation score measured prospectively in a large, initially MS-free population, thereby reducing the potential for reverse causation and providing more robust evidence for the observed association. Beyond methodological considerations, this association is also supported by biological plausibility. Genome-wide analyses by the IMSGC have demonstrated that the majority of MS susceptibility loci implicate genes within innate and adaptive immune pathways (6), and Shams et al. further identified inflammatory signaling mediation, oxidative damage responses, and epigenetic regulation as key contributors to MS genetic susceptibility (38). Collectively, these genetic findings support a plausible biological framework in which chronic peripheral immune dysregulation may interact with underlying genetic architecture to influence MS risk. Nevertheless, whether this interaction reflects a causal pathway or shared upstream determinants warrants further investigation through mechanistic and interventional studies.

The association between LGI and incident MS was significantly modified by socioeconomic deprivation and hypertension, but not by sex, polygenic risk score, or behavioral lifestyle factors (sleep patterns and physical activity), suggesting that peripheral inflammation–driven MS risk may be potentiated by socioeconomic and metabolic factors rather than by genetic predisposition or other lifestyle behaviors. Regarding sex specifically, the incidence density of MS was higher among women than among men in the present study, consistent with the well-documented female predominance in MS (39). However, this sex difference in MS incidence did not translate into a differential effect of systemic inflammation on MS risk, this difference in background susceptibility did not appear to amplify the inflammatory pathway to MS onset. Regarding IMD scores, our analysis revealed that low IMD scores amplified the association between LGI and incident MS, suggesting that socioeconomic factors—such as dietary quality, living conditions, air quality, and other unmeasured environmental exposures—may potentiate the effect of peripheral inflammation on MS development (40, 41). This finding is of particular relevance given the evidence that area-level deprivation also limits access to disease-modifying therapies for MS in England (42), highlighting the need for targeted interventions in socioeconomically disadvantaged populations. Regarding pre-existing cardiometabolic conditions, both hypertension and diabetes appeared to strengthen the LGI–MS association. Mechanistically, endothelial dysfunction likely represents a central shared pathway, given that MS is characterized by significant endothelial impairment, as evidenced by lower reperfusion hyperemia index (RHI) values in patients compared to controls (43). Hypertension may compound this impairment through immune-mediated vascular remodeling, chronic vascular inflammation, and impaired cerebral perfusion (44–47). Furthermore, hypertension-driven oxidative stress can activate matrix metalloproteinases that compromise blood–brain barrier (BBB) integrity (48), thereby facilitating the central infiltration of systemic LGI mediators and potentiating MS-related neuroinflammatory processes. With respect to diabetes, we identified a significant interaction between CRP levels and pre-existing diabetes in relation to MS risk, consistent with prior evidence linking diabetes to elevated MS susceptibility (HR = 1.59) (49). This observation supports the hypothesis that hyperinsulinemia amplifies cytokine responses to inflammatory stimuli, further exacerbating the systemic inflammatory burden relevant to MS pathogenesis (50). Although the observational nature of this study precludes causal inference, these findings suggest that individuals with pre-existing hypertension or diabetes may represent a particularly vulnerable subgroup in whom monitoring and management of systemic low-grade inflammation could be of greater relevance for MS risk mitigation.

Based on the prospective association between LGI and risk of MS, we hypothesized that inflammation-related structural damage to vulnerable brain regions may constitute a key mechanism in MS pathogenesis. To explore this, we leveraged the UK Biobank imaging subsample to examine the associations between LGI and volumetric measures in six brain regions previously reported to undergo atrophy in MS (25–27). Notably, this analysis was conducted in the general imaging cohort, and therefore the results reflect population-level associations rather than MS-specific pathology. Indeed, our results revealed a significant negative association between LGI and the volume of all six MS-related brain regions, lending indirect support to the biological plausibility of this pathway. This finding is consistent with accumulating evidence. Elevated IL-6 and CRP levels have been correlated with reduced gray matter and hippocampal volume as well as increased white matter hyperintensity burden in older adults (51), and a large-scale population study demonstrated that elevated peripheral inflammatory markers are linked to cognitive decline mediated by total gray and white matter atrophy (52). A recent study has also shown a correlation between lipid-related biomarkers and brain atrophy, suggesting potential shared metabolic–inflammatory pathways that could be explored in future research (53). Critically, the fact that LGI was inversely associated with volume in all six regions specifically selected for their known vulnerability in MS—rather than in only a subset—strengthens indirect mechanistic support for the prospective association between LGI and MS risk. This consistency suggests that chronic peripheral inflammation may preferentially target brain regions inherently susceptible to inflammatory insult, potentially lowering the threshold for MS-related neurodegeneration. A previous longitudinal study in progressive MS further supports this directionality, indicating that systemic inflammation temporally precedes cervical cord atrophy (54), although confirmation in brain parenchymal regions requires more prospective MS-specific imaging cohorts.

Furthermore, the inverse associations between LGI and brain structural phenotypes appeared notably more pronounced among male participants. This finding contrasts with the comparable LGI–MS risk observed in both sexes, suggesting that the structural susceptibility of MS-related brain regions to peripheral inflammation may be sex-dependent. A parallel pattern of male vulnerability is observed in the clinical trajectory of MS, where men typically progress to disability milestones more rapidly than women (39) and are more prone to transitioning to secondary progressive MS (55). This progressive phase is characterized by extensive axonal loss and brain atrophy, driven predominantly by compartmentalized inflammatory and neurodegenerative processes within the CNS rather than the peripheral immune-mediated mechanisms that dominate the early relapsing phase (56, 57). Sex differences in microglial reactivity, blood–brain barrier permeability, and the neuroprotective effects of estrogen have all been proposed as potential mechanisms underlying such male vulnerability (58, 59), though these hypotheses remain to be directly tested in future studies. However, we found no significant interaction between MS-PRS and LGI for any brain structural measure, indicating that the inverse association between systemic inflammation and brain volumes in MS-related regions was comparable across different levels of genetic risk. Thus, this relationship does not appear to be limited to genetically susceptible individuals but is broadly observed in the general population, further highlighting the potential importance of monitoring and managing systemic inflammation for brain health at the population level.

Additionally, our decomposition of cortical measures across the three cortical regions revealed that the negative association between LGI and lateral orbitofrontal cortex volume was primarily attributable to smaller surface area, whereas for the supramarginal gyrus and superior temporal gyrus, higher LGI was associated with both lower cortical thickness and smaller surface area. The region-specific patterns observed here suggest that LGI may affect cortical morphology through different mechanisms depending on regional cytoarchitecture and vascular characteristics (60, 61). These population-level findings are consistent with cortical abnormalities documented in the MS literature: lower cortical thickness has been consistently reported in patients, with region-specific reductions correlating with poorer cognitive performance (62), and smaller cortical surface area has been associated with shallower sulcal depth in sensory, cognitive, and motor regions (63). The convergence between these population-level patterns and MS-related cortical pathology suggests that certain cortical regions may be inherently susceptible to both systemic inflammatory insults and MS-related neurodegeneration. Because this study relied on a single imaging time point, prospective studies with serial imaging are needed to clarify the longitudinal impact of LGI on cortical morphology.

Risk stratification strategies identifying individuals with heightened susceptibility are essential for personalized health management and may offer greater efficacy than universal approaches. LGI represents a chronic, subclinical state, due to its insidious nature, LGI often remains undetected during routine clinical assessments, leading to significant gaps in early risk identification despite robust evidence linking this persistent inflammatory burden to the pathogenesis and progression of various chronic conditions (9). Systemic inflammation is shaped by a range of modifiable lifestyle and behavioral factors, including dietary patterns, physical activity levels, sleep adequacy, and psychological wellbeing (64–66). While our observational findings do not permit causal inference, the observed associations between LGI and both MS incidence and unfavorable brain structural phenotypes, coupled with the modifiable nature of many inflammatory determinants, provides a rationale for future interventional studies to examine whether reducing systemic inflammation may favorably influence MS-related outcomes.

This study has several strengths. Leveraging the large-scale UK Biobank cohort with a substantial number of healthy controls to ensure adequate statistical power, we constructed survival models that adjusted for potential confounders and explored mediation pathways linking inflammation to MS. Additionally, we integrated genetic and neuroimaging data to characterize the structural brain correlates associated with inflammation in the context of MS, providing preliminary evidence on potential biological pathways. However, this study also has several limitations. First, the UK Biobank lacks granular MS-specific clinical and radiological data—such as relapse rates, treatment responsiveness, and lesion load. Furthermore, the absence of information on disease progression (primary vs. secondary) limits our ability to explore potentially divergent inflammatory mechanisms across different MS phenotypes. Second, the generalizability of our findings may be limited by the composition of the UK Biobank, which is predominantly composed of individuals of White European ancestry (approximately 97%) and is subject to a recognized ‘healthy volunteer bias’. Furthermore, our analysis was restricted to urban residents. While this exclusion was necessitated to minimize potential confounding from urban-rural lifestyle variations highly correlated with LGI and to ensure the completeness of medical records, it may further restrict the broad applicability of these results to rural or more socioeconomically diverse populations. Third, incident MS cases were identified via hospital inpatient records, which may lack temporal precision regarding symptom onset and potentially lead to delayed case ascertainment. However, the consistency of our primary findings across sensitivity analyses (employing two-year and five-year lag periods to mitigate reverse causality) reinforces the robustness of the observed associations. Fourth, MS cases remaining undiagnosed or managed exclusively in private healthcare settings would not be captured through hospital inpatient linkage, potentially resulting in outcome misclassification that biases associations toward the null. Fifth, due to data access limitations, genetic ancestry principal components and genotyping array were not adjusted for in the PRS model, which may introduce residual population stratification bias. However, the UK Biobank cohort is predominantly of European ancestry, and a sensitivity analysis restricted to White participants (excluding approximately 3% of non-White individuals) yielded consistent results, partially mitigating this concern. Sixth, although brain MRI acquisition (from 2014) postdated the baseline assessment (2006–2010), providing some degree of temporal ordering, the imaging data were cross-sectional. Longitudinal studies with repeated neuroimaging are needed to establish a formal causal relationship between LGI and brain structural changes. Seventh, retrospectively collected confounder data are susceptible to recall bias, and inflammatory markers measured at a single time point may not reflect chronic inflammatory burden. However, the large UK Biobank cohort should mitigate the impact of any resulting non-differential misclassification. Finally, hypertension and diabetes—both linked to MS incidence and MRI measures—typically present later in life than MS; their effects may therefore differ between young-onset and late-onset MS, a distinction our study did not evaluate, and which warrants further investigation.

## 5 Conclusion

In summary, this large-scale prospective cohort study identified that LGI is robustly associated with an increased risk of incident MS. This association was notably amplified by socioeconomic deprivation and cardiometabolic conditions, particularly among individuals with high IMD scores and those with hypertension at baseline, but was not modified by sex, polygenic risk, or lifestyle factors (sleep and physical activity).Furthermore, higher LGI levels were consistently linked to lower volumes across six MS-related brain regions among the study participants, a relationship that appeared more prominent in males but remained independent of individual genetic risk scores. Given the rising prevalence of MS within aging populations, our findings provide valuable longitudinal evidence for risk stratification. While the observational nature of this study precludes definitive causal inference, these results highlight systemic inflammation as a significant correlate of neural integrity and disease susceptibility, warranting further research to determine the potential efficacy of managing subclinical inflammatory states in clinical practice.

## Data Availability

The data used in the present study are available from the UK Biobank with restrictions applied. Access to the UK Biobank data can be requested through a standard protocol (https://www.ukbiobank.ac.uk/register-apply/). This research has been conducted using the UK Biobank Resource under Application Number 99001 (2023-01-31 to 2026-01-31).

## References

[1] Reich DS Lucchinetti CF Calabresi PA . Multiple sclerosis. N Engl J Med. (2018) 378:169–80. doi: 10.1056/NEJMra1401483 29320652 PMC6942519

[2] Graves JS Krysko KM Hua LH Absinta M Franklin RJM Segal BM . Ageing and multiple sclerosis. Lancet Neurol. (2023) 22:66–77. doi: 10.1016/S1474-4422(22)00184-3 36216015

[3] Vaughn CB Jakimovski D Kavak KS Ramanathan M Benedict RHB Zivadinov R . Epidemiology and treatment of multiple sclerosis in elderly populations. Nat Rev Neurol. (2019) 15:329–42. doi: 10.1038/s41582-019-0183-3 31000816

[4] Campbell J Rashid W Cercignani M Langdon D . Cognitive impairment among patients with multiple sclerosis: associations with employment and quality of life. Postgrad Med J. (2017) 93:143–7. doi: 10.1136/postgradmedj-2016-134071 27512050

[5] Musella A Gentile A Rizzo FR De Vito F Fresegna D Bullitta S . Interplay between age and neuroinflammation in multiple sclerosis: effects on motor and cognitive functions. Front Aging Neurosci. (2018) 10:238. doi: 10.3389/fnagi.2018.00238 30135651 PMC6092506

[6] International Multiple Sclerosis Genetics Consortium . Multiple sclerosis genomic map implicates peripheral immune cells and microglia in susceptibility. Science. (2019) 365:eaav7188. doi: 10.1126/science.aav7188 31604244 PMC7241648

[7] Grebenciucova E Berger JR . Immunosenescence: the role of aging in the predisposition to neuro-infectious complications arising from the treatment of multiple sclerosis. Curr Neurol Neurosci Rep. (2017) 17:61. doi: 10.1007/s11910-017-0771-9 28669032

[8] Ajoolabady A Pratico D Vinciguerra M Lip GYH Franceschi C Ren J . Inflammaging: mechanisms and role in the cardiac and vasculature. Trends Endocrinol Metab TEM. (2023) 34:373–87. doi: 10.1016/j.tem.2023.03.005 37076375

[9] Furman D Campisi J Verdin E Carrera-Bastos P Targ S Franceschi C . Chronic inflammation in the etiology of disease across the life span. Nat Med. (2019) 25:1822–32. doi: 10.1038/s41591-019-0675-0 31806905 PMC7147972

[10] Tao Q Ang TFA DeCarli C Auerbach SH Devine S Stein TD . Association of chronic low-grade inflammation with risk of Alzheimer disease in ApoE4 carriers. JAMA Netw Open. (2018) 1:e183597. doi: 10.1001/jamanetworkopen.2018.3597 30646251 PMC6324596

[11] Musubire AK Cheema S Ray JC Hutton EJ Matharu M . Cytokines in primary headache disorders: a systematic review and meta-analysis. J Headache Pain. (2023) 24:36. doi: 10.1186/s10194-023-01572-7 37016284 PMC10071234

[12] Thuraiaiyah J Erritzøe-Jervild M Al-Khazali HM Schytz HW Younis S . The role of cytokines in migraine: a systematic review. Cephalalgia Int J Headache. (2022) 42:1565–88. doi: 10.1177/03331024221118924 35962530

[13] Mongan D Raj Susai S Föcking M Byrne JF Zammit S Cannon M . Associations between plasma inflammatory markers and psychotic disorder, depressive disorder and generalised anxiety disorder in early adulthood: A nested case-control study. Brain, Behavior, and Immunity. (2023) 111:90–100. doi: 10.1016/j.bbi.2023.03.025 37004760

[14] Gialluisi A Costanzo S Castelnuovo AD Bonaccio M Bracone F Magnacca S . Combined influence of depression severity and low-grade inflammation on incident hospitalization and mortality risk in Italian adults. J Affect Disord. (2021) 279:173–82. doi: 10.1016/j.jad.2020.10.004 33059220

[15] Darweesh SKL Wolters FJ Ikram MA de Wolf F Bos D Hofman A . Inflammatory markers and the risk of dementia and Alzheimer’s disease: a meta-analysis. Alzheimers Dement J Alzheimers Assoc. (2018) 14:1450–9. doi: 10.1016/j.jalz.2018.02.014 29605221

[16] Cervellati C Trentini A Bosi C Valacchi G Morieri ML Zurlo A . Low-grade systemic inflammation is associated with functional disability in elderly people affected by dementia. GeroScience. (2018) 40:61–9. doi: 10.1007/s11357-018-0010-6 29428983 PMC5832662

[17] Akil E Alp R Aluclu MU Acar A Kaplan I . Serum endocan levels in multiple sclerosis relapse and remission. Eur Rev Med Pharmacol Sci. (2021) 25:4091–8. doi: 10.26355/eurrev_202106_26051 34156688

[18] Hasselbalch I Søndergaard H Koch-Henriksen N Olsson A Ullum H Sellebjerg F . The neutrophil-to-lymphocyte ratio is associated with multiple sclerosis. Mult Scler J - Exp Transl Clin. (2018) 4:205521731881318. doi: 10.1177/2055217318813183 30515298 PMC6262498

[19] Yetkin MF Mirza M . Neutrophil to-lymphocyte ratio as a possible predictor of prognosis in recently diagnosed multiple sclerosis patients. J Neuroimmunol. (2020) 346:577307. doi: 10.1016/j.jneuroim.2020.577307 32619894

[20] Gelibter S Pisa M Croese T Dalla Costa G Orrico M Preziosa P . Neutrophil-to-lymphocyte ratio: a marker of neuro-inflammation in multiple sclerosis? J Neurol. (2021) 268:717–23. doi: 10.1007/s00415-020-10322-7 33389030

[21] Zhou Q Jia R Dang J . Correlation between the neutrophil-to-lymphocyte ratio and multiple sclerosis: recent understanding and potential application perspectives. Neurol Res Int. (2022) 2022:3265029. doi: 10.1155/2022/3265029 36340639 PMC9629953

[22] Williams JA Burgess S Suckling J Lalousis PA Batool F Griffiths SL . Inflammation and brain structure in schizophrenia and other neuropsychiatric disorders: a Mendelian randomization study. JAMA Psychiatry. (2022) 79:498–507. doi: 10.1001/jamapsychiatry.2022.0407 35353173 PMC8968718

[23] Bao Y Chen X Li Y Yuan S Han L Deng X . Chronic low-grade inflammation and brain structure in the middle-aged and elderly adults. Nutrients. (2024) 16:2313. doi: 10.3390/nu16142313 39064755 PMC11280392

[24] Liu W-S Zhang Y-R Ge Y-J Wang H-F Cheng W Yu J-T . Inflammation and brain structure in Alzheimer’s disease and other neurodegenerative disorders: a Mendelian randomization study. Mol Neurobiol. (2024) 61:1593–604. doi: 10.1007/s12035-023-03648-6 37736795

[25] Pagani E Storelli L Pantano P Petsas N Tedeschi G Gallo A . Multicenter data harmonization for regional brain atrophy and application in multiple sclerosis. J Neurol. (2023) 270:446–59. doi: 10.1007/s00415-022-11387-2 36152049

[26] Lauerer M Bussas M Pongratz V Berthele A Kirschke JS Wiestler B . Percentage brain volume change in multiple sclerosis mainly reflects white matter and cortical volume. Ann Clin Transl Neurol. (2023) 10:130–5. doi: 10.1002/acn3.51700 36427289 PMC9852382

[27] Rocca MA Preziosa P Mesaros S Pagani E Dackovic J Stosic-Opincal T . Clinically isolated syndrome suggestive of multiple sclerosis: dynamic patterns of gray and white matter changes-a 2-year MR imaging study. Radiology. (2016) 278:841–53. doi: 10.1148/radiol.2015150532 26348234

[28] Gialluisi A Bonaccio M Di Castelnuovo A Costanzo S De Curtis A Sarchiapone M . Lifestyle and biological factors influence the relationship between mental health and low-grade inflammation. Brain Behav Immun. (2020) 85:4–13. doi: 10.1016/j.bbi.2019.04.041 31055172

[29] Shi H Schweren LJS Ter Horst R Bloemendaal M van Rooij D Vasquez AA . Low-grade inflammation as mediator between diet and behavioral disinhibition: a UK Biobank study. Brain Behav Immun. (2022) 106:100–10. doi: 10.1016/j.bbi.2022.07.165 35944739

[30] Pounis G Bonaccio M Di Castelnuovo A Costanzo S de Curtis A Persichillo M . Polyphenol intake is associated with low-grade inflammation, using a novel data analysis from the Moli-sani study. Thromb Haemost. (2016) 115:344–52. doi: 10.1160/TH15-06-0487 26355794

[31] Bycroft C Freeman C Petkova D Band G Elliott LT Sharp K . The UK Biobank resource with deep phenotyping and genomic data. Nature. (2018) 562:203–9. doi: 10.1038/s41586-018-0579-z 30305743 PMC6786975

[32] International Multiple Sclerosis Genetics Consortium (IMSGC) . Analysis of immune-related loci identifies 48 new susceptibility variants for multiple sclerosis. Nat Genet. (2013) 45:1353–60. doi: 10.1038/ng.2770 24076602 PMC3832895

[33] Lourida I Hannon E Littlejohns TJ Langa KM Hyppönen E Kuzma E . Association of lifestyle and genetic risk with incidence of dementia. JAMA. (2019) 322:430. doi: 10.1001/jama.2019.9879 31302669 PMC6628594

[34] Li X Zhou T Ma H Huang T Gao X Manson JE . Healthy sleep patterns and risk of incident arrhythmias. J Am Coll Cardiol. (2021) 78:1197–207. doi: 10.1016/j.jacc.2021.07.023 34531019 PMC8454031

[35] Li S Han L Shi H Chong MKC Zhao S Ran J . Excess deaths from Alzheimer’s disease and Parkinson’s disease during the COVID-19 pandemic in the USA. Age Ageing. (2022) 51:afac277. doi: 10.1093/ageing/afac277 36571781

[36] Han L Zhao S Li S Gu S Deng X Yang L . Excess cardiovascular mortality across multiple COVID-19 waves in the United States from March 2020 to March 2022. Nat Cardiovasc Res. (2023) 2:322–33. doi: 10.1038/s44161-023-00220-2 39195997

[37] Chu AL Stochl J Lewis G Zammit S Jones PB Khandaker GM . Longitudinal association between inflammatory markers and specific symptoms of depression in a prospective birth cohort. Brain Behav Immun. (2019) 76:74–81. doi: 10.1016/j.bbi.2018.11.007 30414442 PMC6363967

[38] Shams H Shao X Santaniello A Kirkish G Harroud A Ma Q . Polygenic risk score association with multiple sclerosis susceptibility and phenotype in Europeans. Brain J Neurol. (2023) 146:645–56. doi: 10.1093/brain/awac092 35253861 PMC10169285

[39] Voskuhl RR . The effect of sex on multiple sclerosis risk and disease progression. Mult Scler Houndmills Basingstoke Engl. (2020) 26:554–60. doi: 10.1177/1352458519892491 31965884 PMC7160019

[40] Olsson T Barcellos LF Alfredsson L . Interactions between genetic, lifestyle and environmental risk factors for multiple sclerosis. Nat Rev Neurol. (2017) 13:25–36. doi: 10.1038/nrneurol.2016.187 27934854

[41] Fitzgerald KC Tyry T Salter A Cofield SS Cutter G Fox R . Diet quality is associated with disability and symptom severity in multiple sclerosis. Neurology. (2018) 90:e1–e11. doi: 10.1212/WNL.0000000000004768 29212827

[42] Das J Rog DJ Middleton R Rodgers JW Fry R Nicholas R . The association between deprivation and the access to disease modifying therapies for multiple sclerosis: an England wide community-based study in the UK MS Register. Mult Scler Relat Disord. (2022) 57:103474. doi: 10.1016/j.msard.2021.103474 34986456

[43] Keményová P Siarnik P Sutovský S Blaho A Turcáni P Kollár B . Impairment of endothelial function in patients with multiple sclerosis. Neuro Endocrinol Lett. (2015) 36:67–71. 25789590

[44] Faraco G Iadecola C . Hypertension: a harbinger of stroke and dementia. Hypertens Dallas Tex 1979. (2013) 62:810–7. doi: 10.1161/HYPERTENSIONAHA.113.01063 23980072 PMC3847558

[45] Alexander JS Zivadinov R Maghzi A-H Ganta VC Harris MK Minagar A . Multiple sclerosis and cerebral endothelial dysfunction: mechanisms. Pathophysiol Off J Int Soc Pathophysiol. (2011) 18:3–12. doi: 10.1016/j.pathophys.2010.04.002 20663648

[46] Zhang Z Zhao L Zhou X Meng X Zhou X . Role of inflammation, immunity, and oxidative stress in hypertension: new insights and potential therapeutic targets. Front Immunol. (2022) 13:1098725. doi: 10.3389/fimmu.2022.1098725 36703963 PMC9871625

[47] Sheikh AM Yano S Tabassum S Nagai A . The role of the vascular system in degenerative diseases: mechanisms and implications. Int J Mol Sci. (2024) 25:2169. doi: 10.3390/ijms25042169 38396849 PMC10889477

[48] Bajwa E Klegeris A . Neuroinflammation as a mechanism linking hypertension with the increased risk of Alzheimer’s disease. Neural Regener Res. (2022) 17:2342–6. doi: 10.4103/1673-5374.336869 35535868 PMC9120695

[49] Tang M Tang H Deng H Liu R He G . Diabetes mellitus and risk of multiple sclerosis: a systematic review and meta-analysis. Front Endocrinol. (2026) 17:1724167. doi: 10.3389/fendo.2026.1724167 41788773 PMC12957073

[50] Watson GS Craft S . Insulin resistance, inflammation, and cognition in Alzheimer’s disease: lessons for multiple sclerosis. J Neurol Sci. (2006) 245:21–33. doi: 10.1016/j.jns.2005.08.017 16631207

[51] Satizabal CL Zhu YC Mazoyer B Dufouil C Tzourio C . Circulating IL-6 and CRP are associated with MRI findings in the elderly: the 3C-Dijon Study. Neurology. (2012) 78:720–7. doi: 10.1212/WNL.0b013e318248e50f 22357713

[52] Zhuo B Zheng D Cai M Wang C Zhang S Zhang Z . Mediation effect of brain volume on the relationship between peripheral inflammation and cognitive decline. J Alzheimers Dis Jad. (2023) 95:523–33. doi: 10.3233/JAD-230253 37545239

[53] Chen X Bao Y Zhao J Wang Z Gao Q Ma M . Associations of triglycerides and atherogenic index of plasma with brain structure in the middle-aged and elderly adults. Nutrients. (2024) 16:672. doi: 10.3390/nu16050672 38474800 PMC10933770

[54] Stuart CM Varatharaj A Zou Y Darekar A Domjan J Gandini Wheeler-Kingshott CAM . Systemic inflammation associates with and precedes cord atrophy in progressive multiple sclerosis. Brain Commun. (2024) 6:fcae143. doi: 10.1093/braincomms/fcae143 38712323 PMC11073756

[55] Se D L H Fr P Ma Z . Sex-based differences in multiple sclerosis (part I): biology of disease incidence. Curr Top Behav Neurosci. (2015) 26:29–56. doi: 10.1007/7854_2015_371 25690593

[56] Alvarez-Sanchez N Dunn SE . Immune cell contributors to the female sex bias in multiple sclerosis and experimental autoimmune encephalomyelitis. Curr Top Behav Neurosci. (2023) 62:333–73. doi: 10.1007/7854_2022_324 35467295

[57] Voskuhl RR . The effect of sex on multiple sclerosis risk and disease progression. Multiple Sclerosis Journal. (2020) 26:554–60. doi: 10.1177/1352458519892491 31965884 PMC7160019

[58] Vegeto E Benedusi V Maggi A . Estrogen anti-inflammatory activity in brain: a therapeutic opportunity for menopause and neurodegenerative diseases. Front Neuroendocrinol. (2008) 29:507–19. doi: 10.1016/j.yfrne.2008.04.001 18522863 PMC2630539

[59] Villa A Gelosa P Castiglioni L Cimino M Rizzi N Pepe G . Sex-specific features of microglia from adult mice. Cell Rep. (2018) 23:3501–11. doi: 10.1016/j.celrep.2018.05.048 29924994 PMC6024879

[60] Zhao Z Nelson AR Betsholtz C Zlokovic BV . Establishment and dysfunction of the blood-brain barrier. Cell. (2015) 163:1064–78. doi: 10.1016/j.cell.2015.10.067 26590417 PMC4655822

[61] Amunts K Zilles K . Architectonic mapping of the human brain beyond Brodmann. Neuron. (2015) 88:1086–107. doi: 10.1016/j.neuron.2015.12.001 26687219

[62] Stellmann J-P Wanke N Maarouf A Gellißen S Heesen C Audoin B . Cognitive performance shows domain specific associations with regional cortical thickness in multiple sclerosis. NeuroImage Clin. (2021) 30:102606. doi: 10.1016/j.nicl.2021.102606 33744503 PMC7985400

[63] Genç B Aslan K Şen S İncesu L . Cortical morphological changes in multiple sclerosis patients: a study of cortical thickness, sulcal depth, and local gyrification index. Neuroradiology. (2023) 65:1405–13. doi: 10.1007/s00234-023-03185-y 37344675

[64] Faubel N Makran M Barberá R Garcia-Llatas G Giardina IC Tesoriere L . Anti-inflammatory activity of plant sterols in a co-culture model of intestinal inflammation: focus on food-matrix effect. Food Funct. (2024) 15:6502–11. doi: 10.1039/D4FO00917G 38804902

[65] Grosso G Laudisio D Frias-Toral E Barrea L Muscogiuri G Savastano S . Anti-inflammatory nutrients and obesity-associated metabolic-inflammation: state of the art and future direction. Nutrients. (2022) 14:1137. doi: 10.3390/nu14061137 35334794 PMC8954840

[66] Suzuki K . Chronic inflammation as an immunological abnormality and effectiveness of exercise. Biomolecules. (2019) 9:223. doi: 10.3390/biom9060223 31181700 PMC6628010

