# Supplementary material for: Chronic low-grade inflammation, genetic susceptibility, and the risk of multiple sclerosis: a prospective cohort study
Source: Front Immunol. 2026 Aug 26;17:1805464. doi: 10.3389/fimmu.2026.1805464 (PMC13553424; doi:10.3389/fimmu.2026.1805464)
Supplement: Supplementary file 1 [file DataSheet1.pdf]

Supplementary Documents

Chronic low-grade inflammation, genetic susceptibility, and the incidence risk of multiple sclerosis: A prospective cohort study

eMethods 2
Figure S1. Definition, exclusion and history of INFLA-score 3
Table S1. Definition of INFLA-score 4
Table S2. Definition of primary and secondary outcomes 5
Table S3. SNPs used to build the genetic risk score for MS 6
Table S4. Healthy lifestyle factor definitions 9
Table S5. Incidence density of multiple sclerosis among different populations 11
Table S6. Associations of the incidence risk of multiple sclerosis with low-grade inflammation .. 12
Table S7. Associations of the incidence risk of multiple sclerosis with the inflammation biomarkers of low-grade inflammation 13
Table S8. Sensitivity analysis of the main associations 14
Table S9. Associations of low-grade inflammation and the inflammation biomarkers with incidence risks of multiple sclerosis across healthy lifestyle subgroups 16
Table S10. Associations of low-grade inflammation and the inflammation biomarkers with the brain structures 17
Table S11. Associations between the brain structures and low-grade inflammation–across sex subgroups 19
Table S12. Associations of the brain structures with CRP and WBC across sex subgroups 20
Table S13. Associations of the brain structures with PLT and NLR across sex subgroups 21
Table S14 Associations between the brain structures and low-grade inflammation-across genetic risk subgroups 22

eMethods

Brain magnetic resonance imaging

Neuroimaging data were collected on a standard Siemens Skyra 3T scanner with a 32-channel head coil. Quality-controlled T1-weighted neuroimaging data were processed with FreeSurfer, surface templates were utilized to extract imaging derived phenotypes referring to atlas regions' surface volume. Any FreeSurfer outputs that failed to pass quality control were excluded from the FreeSurfer imaging-derived phenotypes. Qoala-T approach was used to check FreeSurfer outputs, supplemented by manual checking of outputs close to threshold. The Subcortical volumes (FIRST) (category 1102) and FreeSurfer desikan white (category 192) correspond to 7 subcortical regions and 33 cortical regions.

Blood sample processing

Blood cell counts were performed using a Beckman Coulter LH750 to determine the total number of leukocytes, platelets, neutrophils and lymphocytes. Serum C-reactive protein (CRP) was measured by immunoturbidimetric high-sensitivity analysis on a Beckman Coulter AU5800. Details regarding the blood sample processing can be found at https://biobank.ndph.ox.ac.uk/showcase/label.cgi?id=100080.

Polygenic risk score (PRS)

The procedure for genotyping and imputation in the UK Biobank has been described in detail previously[1]. To construct the genetic risk score (PRS), we used 110 single nucleotide polymorphisms (SNPs) reported by the International Multiple Sclerosis Genetics Consortium (IMSGC)[2]. All 110 SNPs needed for our analysis were available in the UK Biobank imputed database. According to the number of risk alleles, each SNP was recoded as 0, 1 or 2 according to the number of risk estimate (β coefficient) on MS. The PRS was calculated using the equation:PRS $= \sum_{j=1}^{M} \beta_j \times SNP_j$, where M is the total number of SNPs, and β_j is per allele log odds ratio (OR) for lung cancer associated with SNP_j. Then participants were categorized into three groups: low (lowest tertile), intermediate (intermediate tertile), or high (highest tertile) genetic risk.

Reference

1. Bycroft C, Freeman C, Petkova D, et al. The UK Biobank resource with deep phenotyping and genomic data. *Nature* 2018; 562: 203–209.
2. International Multiple Sclerosis Genetics Consortium (IMSGC). Analysis of immune-related loci identifies 48 new susceptibility variants for multiple sclerosis. *Nat Genet* 2013; 45: 1353–1360.

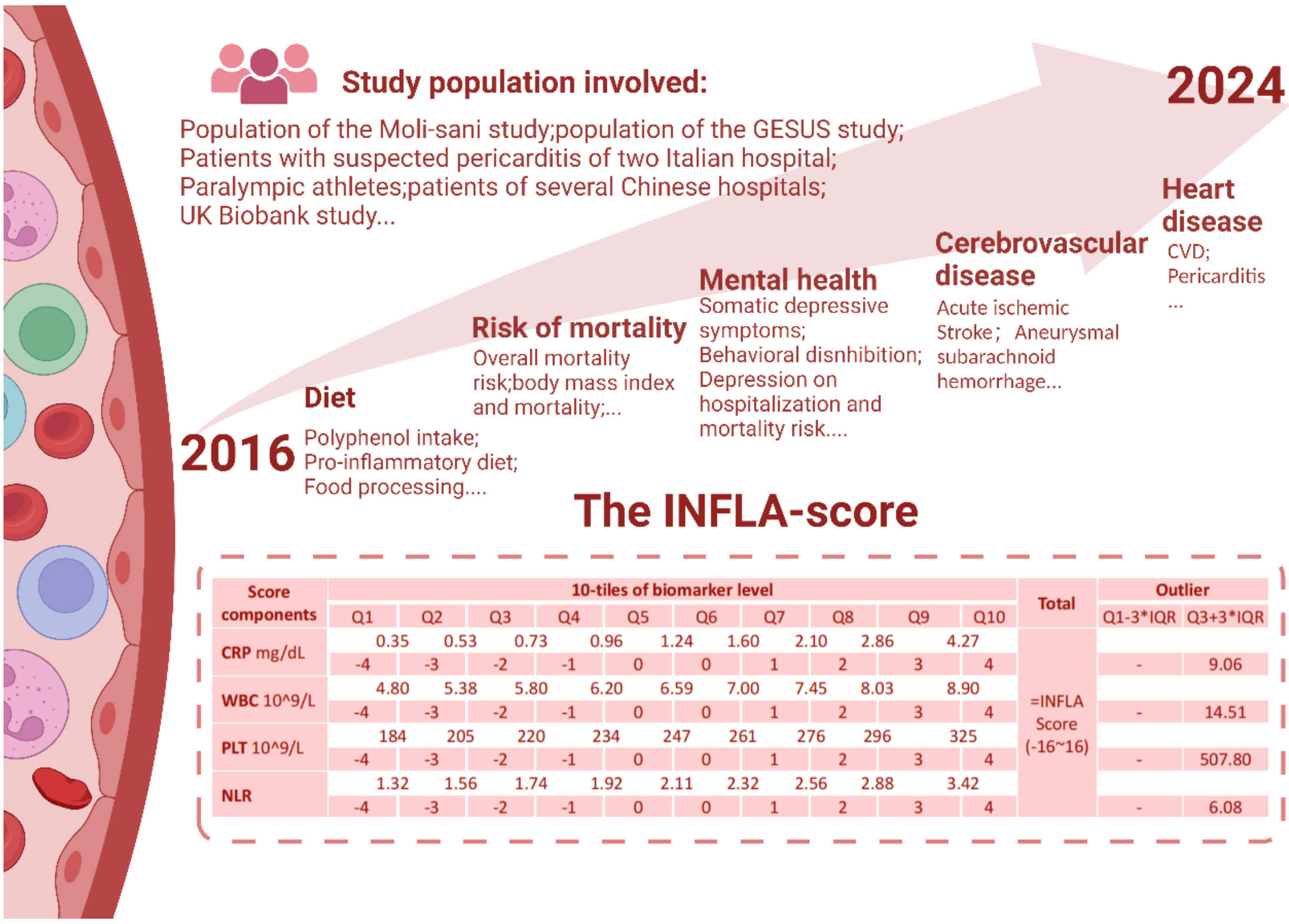

Score components	10-tiles of biomarker level										Total	Outlier	
	Q1	Q2	Q3	Q4	Q5	Q6	Q7	Q8	Q9	Q10		Q1-3*IQR	Q3+3*IQR
CRP mg/dL	0.35	0.53	0.73	0.96	1.24	1.60	2.10	2.86	4.27		=INFLA Score (-16~16)		
	-4	-3	-2	-1	0	0	1	2	3	4		-	9.06
WBC 10^9/L	4.80	5.38	5.80	6.20	6.59	7.00	7.45	8.03	8.90				
	-4	-3	-2	-1	0	0	1	2	3	4		-	14.51
PLT 10^9/L	184	205	220	234	247	261	276	296	325				
	-4	-3	-2	-1	0	0	1	2	3	4		-	507.80
NLR	1.32	1.56	1.74	1.92	2.11	2.32	2.56	2.88	3.42				
	-4	-3	-2	-1	0	0	1	2	3	4		-	6.08

Figure S1. Definition, exclusion and history of INFLA-score

Table S1. Definition of INFLA-score

INFLA-score		Field code
C-reactive protein	/	30710
White blood cell (leukocyte) count	/	30000
Platelet count	/	30080
Neutrophil to Lymphocyte ratio	Neutrophil count	30140
	Lymphocyte count	30120

Table S2. Definition of primary and secondary outcomes

Outcomes	Abbr	Unit	Source
**Primary outcomes**			
Multiple sclerosis	MS	\	ICD 9: 340, ICD 10: G35
**Secondary outcomes**			
**Volume of subcortical structure**			
Caudate nucleus	CN	mm^3	UKB data field 25013 and 25014
Putamen	PUT	mm^3	UKB data field 25015 and 25016
Thalamus	THA	mm^3	UKB data field 25011 and 25012
**Volume of cortical structure**			
Lateral orbitofrontal cortex	lOFC	mm^3	UKB data field 26799 and 26900
Supramarginal gyrus	SMG	mm^3	UKB data field 26818 and 26919
Superior temporal gyrus	STG	mm^3	UKB data field 26817 and 26918
**Area of cortical structure**			
Lateral orbitofrontal cortex	lOFC	mm^2	UKB data field 26732 and 26833
Supramarginal gyrus	SMG	mm^2	UKB data field 26751 and 26852
Superior temporal gyrus	STG	mm^2	UKB data field 26750 and 26851
**Mean thickness of cortical structure**			
Lateral orbitofrontal cortex	lOFC	mm	UKB data field 26766 and 26867
Supramarginal gyrus	SMG	mm	UKB data field 26785 and 26886
Superior temporal gyrus	STG	mm	UKB data field 26784 and 26885

Table S3. SNPs used to build the genetic risk score for MS

No.	SNP rsID	Chr	Position	Effect Allele
1	rs3748817	1	2525665	A
2	rs3007421	1	6530189	A
3	rs12087340	1	85746993	A
4	rs11587876	1	85915183	A
5	rs41286801	1	92975464	A
6	rs7552544	1	101240893	A
7	rs11581062	1	101407519	G
8	rs6677309	1	117080166	A
9	rs666930	1	120258970	G
10	rs2050568	1	157770241	G
11	rs35967351	1	160711804	A
12	rs1359062	1	192541472	C
13	rs55838263	1	200874728	A
14	rs4665719	2	25017860	G
15	rs2163226	2	43361256	A
16	rs842639	2	61095245	A
17	rs7595717	2	68587477	A
18	rs17174870	2	112665201	G
19	rs9967792	2	191974435	G
20	rs9989735	2	231115454	C
21	rs11719975	3	18785585	C
22	rs2371108	3	27757018	A
23	rs1813375	3	28078571	A
24	rs4679081	3	33013483	G
25	rs9828629	3	71530346	G
26	rs2028597	3	105558837	G
27	rs1131265	3	119222456	C
28	rs1920296	3	121543577	C
29	rs2255214	3	121770539	C
30	rs9282641	3	121796768	G
31	rs1014486	3	159691112	G
32	rs7665090	4	103551603	G
33	rs2726518	4	106173199	C
34	rs6881706	5	35879156	C
35	rs6880778	5	40399096	G
36	rs71624119	5	55440730	G
37	rs756699	5	133446575	A
38	rs371522651	5	141506564	C
39	rs2546890	5	158759900	A
40	rs4976646	5	176788570	G
41	rs17119	6	14719496	A
42	rs941816	6	36375304	G
43	rs72928038	6	90976768	A
44	rs802734	6	128278798	A
45	rs11154801	6	135739355	A
46	rs17066096	6	137452908	G
47	rs7769192	6	137962655	G

48	rs67297943	6	138244816	A
49	rs212405	6	159470559	T
50	rs1843938	7	3113034	A
51	rs706015	7	27014988	C
52	rs917116	7	28172739	C
53	rs60600003	7	37382465	C
54	rs201847125	7	50325567	G
55	rs354033	7	149289464	G
56	rs1021156	8	79575804	A
57	rs2456449	8	128192981	G
58	rs4410871	8	128815029	G
59	rs759648	8	129158945	C
60	rs2150702	9	5893861	G
61	rs2104286	10	6099045	A
62	rs793108	10	31415106	A
63	rs2688608	10	75658349	A
64	rs1782645	10	81048611	A
65	rs7923837	10	94481917	G
66	rs7120737	11	47702395	G
67	rs34383631	11	60793330	A
68	rs694739	11	64097233	A
69	rs533646	11	118566746	G
70	rs9736016	11	118724894	T
71	rs523604	11	118755738	A
72	rs1800693	12	6440009	G
73	rs12296430	12	6503500	C
74	rs11052877	12	9905690	G
75	rs201202118	12	58182062	A
76	rs7132277	12	123593382	A
77	rs4772201	13	100086259	A
78	rs2236262	14	69261472	A
79	rs4903324	14	75961511	A
80	rs74796499	14	88432328	C
81	rs12148050	14	103263788	A
82	rs59772922	15	79207466	A
83	rs8042861	15	90977333	A
84	rs2744148	16	1073552	G
85	rs12927355	16	11194771	G
86	rs4780346	16	11288806	A
87	rs6498184	16	11435990	G
88	rs7204270	16	30156963	G
89	rs1886700	16	68685905	A
90	rs12149527	16	79110596	A
91	rs7196953	16	79649394	A
92	rs35929052	16	85994484	G
93	rs12946510	17	37912377	A
94	rs4796791	17	40530763	A
95	rs4794058	17	45597098	A
96	rs8070345	17	57816757	A

97	rs7238078	18	56384192	A
98	rs1077667	19	6668972	G
99	rs34536443	19	10463118	C
100	rs2288904	19	10742170	G
101	rs1870071	19	16505106	G
102	rs11554159	19	18285944	G
103	rs8107548	19	49870643	G
104	rs4810485	20	44747947	A
105	rs17785991	20	48438761	A
106	rs2248359	20	52791518	G
107	rs2256814	20	62373983	A
108	rs6062314	20	62409713	A
109	rs2283792	22	22131125	C
110	rs470119	22	50966914	A

Table S4. Healthy lifestyle factor definitions

Demographic variables	Source and definition	Self-reported UK Biobank field code
Index of multiple deprivation scores	Obtained from local NHS Primary Care Trust registries at baseline; This index come from a UK government qualitative study of deprived areas in British local councils, IMD scores are calculated by 8 to 9 scores from crime score, education score, employment score, health score, housing score, income score, living environment score, access to services score, community safety score and physical environment score, in different ways between England, Scotland and Wales. IMD scores was categorized into low and high levels by the median.	26410, 26427, 26426
Waist-to-hip ratio	UK Biobank physical measurements at baseline; This field was calculated through waist circumference in centimeters divided by hip circumference in centimeters and classified into ideal and poor categories with 0.85 for women and 0.90 for men as the cutoff value.	48 (waist circumference), 49 (hip circumference)
**Healthy lifestyle factor**	**Source and definition**	**Self-reported UK Biobank field code**
Never smoking	UK Biobank Touchscreen questionnaire at baseline; This field summarized the current/past smoking status of the participant, divided into current, previous, and never smoker.<br>Yes: never smoking, No: current and previous smoking	20116
Never drinking	UK Biobank Touch screen questionnaire at baseline; This field summarized the current/past: alcohol drinker status of the participant, divided into current, previous, and never drinker.<br>Yes: never drinking; No: current and previous drinking.	20117
Regular physical activity	UK Biobank Touchscreen questionnaire at baseline; ≥150 minutes moderate activity per week OR ≥ 75 minutes vigorous activity per week OR equivalent combination OR moderate physical activity at least 5 days a week or vigorous activity once a week	22035
Healthy diet	UK Biobank Food Frequency Questionnaire at baseline; At least 4 of the following 7 food groups: 1. Fruits: ≥ 3 servings/day 2. Vegetables: ≥ 3 servings/day 3. Fish: ≥2 servings/week 4. Processed meats: ≤ 1 serving/week 5. Unprocessed red meats: ≤ 1.5 servings/week 6. Whole grains: ≥ 3servings/day 7. Refined grains: ≤1.5servings/day	1309, 1319, 1289, 1299, 1329, 1339, 1349, 1369, 1379, 1389, 1438, 1448, 1458, 1468
Sleep healthy scores	UK Biobank Touchscreen questionnaire at baseline：<br>Chronotype was assessed by the question, "Do you consider yourself to be: 1) definitely a 'morning' person; 2) more a 'morning' person than 'evening' person; 3) more an 'evening' person than a 'morning' person; 4) definitely an 'evening' person." Sleep duration was reported as the hours of sleep in every 24 hours (including naps). Insomnia symptoms were obtained by the question, "Do you have trouble falling asleep at night or do you wake up in the middle of the night?" with multiple choices provided: 1) never/rarely; 2) sometimes; and 3) usually. Snoring was asked by the question "Does your partner close relative or friend complain about your	1180<br>1160<br>1200<br>1210<br>1220

	snoring?" with responses: 1) yes; or 2) no. Daytime sleepiness was asked by the question, "How likely are you to doze off or fall asleep during the daytime when you don't mean to? (eg, when working, reading or driving)" with the following choices: 1) never/rarely; 2) sometimes; 3) often; and 4) all of the time.<br>An index score for the healthy sleep pattern was created including 5 aspects of sleep behaviors: chronotype, sleep duration, insomnia, snoring, and excessive daytime sleepiness. Healthy sleep factors were defined as early chronotype ("morning" or "morning than evening"); sleep 7-8 h/day; reported never/rarely or sometimes insomnia symptoms; no self-reported snoring; and no excessive daytime sleepiness ("never/rarely" or "sometimes"). Each sleep factor was coded 1 if meeting the healthy criterion and 0 if not. The index score for the healthy sleep pattern was obtained by summing up the 5 sleep factors. A higher score indicates a healthier sleep pattern. We also categorized it into "healthy sleep pattern" (healthy sleep score $\geq$ 4), "intermediate sleep pattern" (2 $\leq$ healthy sleep score $\leq$ 3), and "poor sleep pattern" (healthy sleep score $\leq$ 1).	

Table S5. Incidence density of multiple sclerosis among different populations

Subgroup	Number of the incidence case	Total person year	Incidence density (/100,000)
All cohort	432	5,081,861	8.5
Male	134	2,314,179	5.8
Female	298	2,767,682	10.8
Low genetic risk	103	1,698,807	6.1
Moderate genetic risk	147	1,693,427	8.7
High genetic risk	182	1,688,769	10.8

Table S6. Associations of the incidence risk of multiple sclerosis with low-grade inflammation

Models	High-level LGI[a]	
	HR (95% CI)	*P* value
Model 1[b]	1.54 (1.27, 1.87)	<0.001
Model 2[c]	1.50 (1.24, 1.82)	<0.001
Model 3[d]	1.38 (1.10, 1.72)	0.005

Abbreviation: LGI, low-grade inflammation; PRS, polygenic risk scores; HR, hazard ratios; CI, confidence intervals

a The reference is low-level LGI.

b Modal 1 did not adjust for any variables.

c Model 2 adjusted for age, sex and index of multiple deprivation scores.

d Model 3 adjusted for age, sex, index of multiple deprivation scores, waist-to-hip ratio, no smoking, no drinking, regular physical activity, healthy sleep pattern, healthy diet, and chronic disease condition including hypertension, diabetes and stroke.

Table S7. Associations of the incidence risk of multiple sclerosis with the inflammation biomarkers of low-grade inflammation

Inflammation biomarkers	High-level LGI[a]	
	HR (95% CI)	*P* value
CRP		
Model 1[b]	1.22 (1.01, 1.47)	0.042
Model 2[c]	1.20 (0.99, 1.45)	0.059
Model 3[d]	1.09 (0.87, 1.36)	0.462
WBC		
Model 1	1.47 (1.21, 1.77)	<0.001
Model 2	1.47 (1.22, 1.78)	<0.001
Model 3	1.41 (1.13, 1.76)	0.002
PLT		
Model 1	1.39 (1.15, 1.67)	0.001
Model 2	1.23 (1.02, 1.49)	0.034
Model 3	1.37 (1.10, 1.71)	0.005
NRL		
Model 1	1.41 (1.17, 1.70)	<0.001
Model 2	1.49 (1.24, 1.81)	<0.001
Model 3	1.42 (1.14, 1.76)	0.001

Abbreviation: LGI, low-grade inflammation; HR, hazard ratios; CI, confidence intervals; CRP, C-reactive protein; WBC, white blood cell; PLT, platelets; NLR, neutrophil to lymphocyte ratio;

a The reference is low-level of each inflammation biomarkers.

b Modal 1 did not adjust for any variables.

c Model 2 adjusted for age, sex and index of multiple deprivation scores.

d Model 3 adjusted for age, sex, index of multiple deprivation scores, waist-to-hip ratio, no smoking, no drinking, regular physical activity, healthy sleep pattern, healthy diet, and chronic disease condition including hypertension, diabetes and stroke.

Table S8. Sensitivity analysis of the main associations

Sensitivity analysis	High-level LGI		Sex			
			Male		Female	
	HR (95% CI)	*P* value	HR (95% CI)	*P* value	HR (95% CI)	*P* value
Sensitivity analysis 1[a]						
LGI	1.36 (1.08, 1.70)	0.008	1.21 (0.83, 1.78)	0.326	1.43 (1.08, 1.89)	0.012
CRP	1.09 (0.87, 1.37)	0.46	1.02 (0.69, 1.52)	0.902	1.12 (0.84, 1.48)	0.432
WBC	1.43 (1.14, 1.78)	0.002	1.71 (1.16, 2.52)	0.007	1.30 (0.99, 1.71)	0.059
PLT	1.35 (1.08, 1.69)	0.008	1.40 (0.95, 2.07)	0.085	1.32 (1.01, 1.74)	0.042
NLR	1.40 (1.12, 1.74)	0.003	1.37 (0.94, 2.00)	0.102	1.40 (1.07, 1.84)	0.014
Sensitivity analysis 2[b]						
LGI	1.35 (1.07, 1.70)	0.011	1.14 (0.77, 1.71)	0.509	1.45 (1.10, 1.93)	0.009
CRP	1.14 (0.9, 1.44)	0.269	1.12 (0.75, 1.68)	0.58	1.15 (0.86, 1.53)	0.346
WBC	1.43 (1.14, 1.80)	0.002	1.66 (1.11, 2.48)	0.013	1.33 (1.00, 1.75)	0.047
PLT	1.33 (1.06, 1.67)	0.014	1.26 (0.84, 1.89)	0.271	1.37 (1.03, 1.80)	0.028
NLR	1.43 (1.14, 1.79)	0.002	1.34 (0.91, 1.98)	0.144	1.46 (1.11, 1.93)	0.007
Sensitivity analysis 3[c]						
LGI	1.38 (1.10, 1.72)	0.005	1.27 (0.87, 1.86)	0.216	1.43 (1.09, 1.88)	0.010
CRP	1.09 (0.87, 1.37)	0.439	1.06 (0.72, 1.56)	0.767	1.11 (0.84, 1.46)	0.469
WBC	1.41 (1.13, 1.76)	0.002	1.67 (1.14, 2.44)	0.009	1.30 (0.99, 1.70)	0.059
PLT	1.37 (1.10, 1.71)	0.005	1.47 (1.01, 2.15)	0.047	1.32 (1.01, 1.73)	0.039
NLR	1.42 (1.14, 1.76)	0.002	1.41 (0.97, 2.05)	0.070	1.41 (1.08, 1.84)	0.011
Sensitivity analysis 4[d]						
LGI	1.38 (1.10, 1.72)	0.005	1.27 (0.87, 1.86)	0.216	1.43 (1.09, 1.88)	0.010
CRP	0.94 (0.72, 1.24)	0.671	0.93 (0.59, 1.44)	0.732	0.95 (0.67, 1.35)	0.774
WBC	1.41 (1.13, 1.76)	0.002	1.67 (1.14, 2.44)	0.009	1.30 (0.99, 1.70)	0.059
PLT	1.37 (1.10, 1.71)	0.005	1.47 (1.01, 2.15)	0.047	1.32 (1.01, 1.73)	0.039
NLR	1.42 (1.14, 1.76)	0.002	1.41 (0.97, 2.05)	0.070	1.41 (1.08, 1.84)	0.011
Sensitivity analysis 5[e]						
LGI	1.38 (1.10, 1.72)	0.005	1.27 (0.87, 1.86)	0.216	1.43 (1.09, 1.88)	0.010
CRP	0.98 (0.72, 1.34)	0.913	0.94 (0.57, 1.53)	0.791	1.01 (0.68, 1.5)	0.969

WBC	1.41 (1.13, 1.76)	0.002	1.67 (1.14, 2.44)	0.009	1.30 (0.99, 1.70)	0.059
PLT	1.37 (1.10, 1.71)	0.005	1.47 (1.01, 2.15)	0.047	1.32 (1.01, 1.73)	0.039
NLR	1.42 (1.14, 1.76)	0.002	1.41 (0.97, 2.05)	0.070	1.41 (1.08, 1.84)	0.011

Abbreviation: LGI, low-grade inflammation; HR, hazard ratio; CI, confidence intervals; CRP, C-reactive protein; WBC, white blood cell; PLT, platelets; NLR, neutrophil to lymphocyte ratio;

a The cohort was restricted to individuals with White European ancestry, as non-White participants only constitute approximately 3% of the dataset.

b Due to the effects of the COVID-19 pandemic, the follow-up deadline was moved forward to December 31, 2019.

c The covariate of never drinking was changed to moderate drinking.

d Individuals who developed multiple sclerosis within two years of the baseline assessment were excluded from the study.

e Individuals who developed multiple sclerosis within five years of the baseline assessment were excluded from the study.

Table S9. Associations of low-grade inflammation and the inflammation biomarkers with incidence risks of multiple sclerosis across healthy lifestyle subgroups

Subgroup	LGI[a]		CRP[b]		WBC[c]		PLT[d]		NLR[e]	
	HR (95% CI)	*p* for interaction	HR (95% CI)	*p* for interaction	HR (95% CI)	*p* for interaction	HR (95% CI)	*p* for interaction	HR (95% CI)	*p* for interaction
Healthy sleep pattern[f]										
Yes	1.32 (0.98, 1.77)	0.664	1.09 (0.80, 1.47)	0.981	1.31 (0.97, 1.76)	0.463	1.27 (0.95, 1.71)	0.472	1.54 (1.15, 2.06)	0.435
No	1.45 (1.05, 2.01)		1.09 (0.79, 1.51)		1.54 (1.12, 2.12)		1.54 (1.12, 2.12)		1.29 (0.94, 1.78)	
Regular physical activity[g]										
Yes	1.24 (0.91, 1.70)	0.376	0.91 (0.65, 1.27)	0.152	1.26 (0.92, 1.73)	0.397	1.24 (0.91, 1.70)	0.397	1.46 (1.06, 1.99)	0.834
No	1.51 (1.11, 2.05)		1.26 (0.93, 1.71))		1.56 (1.16, 2.11)		1.50 (1.11, 2.02))		1.39 (1.03, 1.87)	

Abbreviation: LGI, low-grade inflammation; HR, hazard ratios; CI, confidence intervals; CRP, C-reactive protein; WBC, white blood cell; PLT, platelets; NLR, neutrophil to lymphocyte ratio; IMD scores, index of multiple deprivation scores

a The reference is low-level LGI.

b The reference is low-level CRP.

c The reference is low-level WBC.

d The reference is low-level PLT.

e The reference is low-level NLR.

f Healthy sleep pattern was defined as healthy sleep score ≥4, and healthy sleep score include 5 aspects: chronotype, sleep duration, insomnia, snoring, and excessive daytime sleepiness, healthy sleep factors were defined as early chronotype ("morning" or "morning than evening"); sleep 7-8 h/day; reported never/rarely or sometimes insomnia symptoms; no self-reported snoring; and no excessive daytime sleepiness ("never/rarely" or "sometimes"). Each sleep factor was coded 1 if meeting the healthy criterion and 0 if not.

g Regular physical activity was defined as meeting the American Heart Association recommendations of at least 150 minutes of moderate activity per week or 75 minutes of vigorous activity per week (or an equivalent combination) or engaging in moderate physical activity at least 5 days a week or vigorous activity once a week.

Table S10. Associations of low-grade inflammation and the inflammation biomarkers with the brain structures

Brain region	LGI[a]			CRP[b]			WBC[c]			PLT[d]			NLR[e]		
	β (95% CI)	*P* value	*P* value FDR[f]	*β* (95% CI)	*P* value	*P* value FDR	*β* (95% CI)	*P* value	*P* value FDR	*β* (95% CI)	*P* value	*P* value FDR	*β* (95% CI)	*P* value	*P* value FDR
Volume of subcortical structure															
CN	-0.041 (-0.064, -0.017)	0.001	0.002	-0.049 (-0.074, -0.025)	<0.001	<0.001	-0.061 (-0.085, -0.038)	<0.001	<0.001	0.001 (-0.023, 0.025)	0.933	0.933	0.001 (-0.022, 0.025)	0.903	0.918
PUT	-0.047 (-0.069, -0.025)	<0.001	<0.001	-0.051 (-0.074, -0.028)	<0.001	<0.001	-0.058 (-0.080, -0.036)	<0.001	<0.001	-0.012 (-0.034, 0.010)	0.298	0.447	0.002 (-0.020, 0.024)	0.859	0.918
THA	-0.057 (-0.079, -0.035)	<0.001	<0.001	-0.087 (-0.110, -0.064)	<0.001	<0.001	-0.046 (-0.068, -0.023)	<0.001	<0.001	-0.019 (-0.041, 0.003)	0.097	0.171	-0.003 (-0.025, 0.019)	0.779	0.850
Volume of cortical structure															
lOFC	-0.050 (-0.072, -0.027)	<0.001	<0.001	-0.059 (-0.082, -0.035)	<0.001	<0.001	-0.053 (-0.076, -0.031)	<0.001	<0.001	-0.003 (-0.026, 0.019)	0.764	0.849	-0.001 (-0.023, 0.021)	0.902	0.918
SMG	-0.036 (-0.058, -0.013)	0.002	0.004	-0.048 (-0.072, -0.025)	<0.001	<0.001	-0.034 (-0.057, -0.011)	0.004	0.008	-0.008 (-0.030, 0.015)	0.513	0.693	-0.002 (-0.024, 0.020)	0.877	0.918
STG	-0.053 (-0.076, -0.031)	<0.001	<0.001	-0.059 (-0.083, -0.036)	<0.001	<0.001	-0.062 (-0.085, -0.039)	<0.001	<0.001	-0.014 (-0.036, 0.009)	0.237	0.365	-0.006 (-0.029, 0.016)	0.567	0.724
Mean thickness of cortical structure															
lOFC	-0.020 (-0.045, 0.004)	0.108	0.180	-0.038 (-0.064, -0.013)	0.003	0.006	-0.011 (-0.036, 0.014)	0.387	0.553	0.006 (-0.019, 0.030)	0.658	0.794	0.005 (-0.019, 0.029)	0.675	0.794
SMG	-0.024 (-0.048, -0.001)	0.048	0.090	-0.035 (-0.060, -0.010)	0.006	0.012	0.008 (-0.016, 0.032)	0.520	0.693	-0.012 (-0.036, 0.012)	0.312	0.457	0.004 (-0.020, 0.027)	0.760	0.849
STG	-0.039 (-0.062, -0.015)	0.001	0.002	-0.064 (-0.089, -0.039)	<0.001	<0.001	-0.016 (-0.041, 0.008)	0.195	0.308	-0.017 (-0.041, 0.007)	0.164	0.266	0.020 (-0.004, 0.043)	0.102	0.175
Area of cortical structure															
lOFC	-0.042 (-0.064, -0.020)	<0.001	<0.001	-0.043 (-0.066, -0.020)	<0.001	<0.001	-0.049 (-0.071, -0.026)	<0.001	<0.001	-0.006 (-0.029, 0.016)	0.573	0.724	-0.006 (-0.028, 0.016)	0.579	0.724
SMG	-0.028 (-0.050, -0.006)	0.014	0.027	-0.040 (-0.063, -0.017)	0.001	0.002	-0.038 (-0.061, -0.015)	0.001	0.002	-0.004 (-0.026, 0.019)	0.749	0.849	-0.005 (-0.027, 0.017)	0.664	0.794
STG	-0.045 (-0.067, -0.023)	<0.001	<0.001	-0.040 (-0.063, -0.017)	0.001	0.002	-0.063 (-0.085, -0.040)	<0.001	<0.001	-0.009 (-0.032, 0.013)	0.400	0.558	-0.021 (-0.042, 0.001)	0.062	0.113

Abbreviation: LGI, low-grade inflammation; CI, confidence intervals; CRP, C-reactive protein; WBC, white blood cell (leukocyte) count; PLT, platelet count; NLR,

neutrophil to lymphocyte ratio; FDR, false discovery rate; CN, caudate nucleus; PUT, putamen; THA, thalamus; lOFC, lateral orbitofrontal cortex; SMG, supramarginal gyrus; STG, superior temporal gyrus

a The reference is low-level LGI.

b The reference is low-level CRP.

c The reference is low-level WBC.

d The reference is low-level PLT.

e The reference is low-level NLR.

f The '*P* value FDR' is a measure of the error detection rate obtained by correcting for the difference significance P value. A low 'P value FDR value' (typically less than 0.05) suggests statistical significance.

Table S11. Associations between the brain structures and low-grade inflammation-across sex subgroups

Phenotype	β (95% CI)[a]		*P* for interaction	*P* value FDR[b]
	Male	Female		
Volume of subcortical structure				
CN	-0.048 (-0.082, -0.015)	-0.034 (-0.066, -0.002)	0.539	0.620
PUT	-0.077 (-0.109, -0.046)	-0.020 (-0.050, 0.009)	0.009	0.036
THA	-0.088 (-0.120, -0.057)	-0.030 (-0.060, 0.000)	0.008	0.034
Volume of cortical structure				
lOFC	-0.080 (-0.112, -0.048)	-0.023 (-0.053, 0.008)	0.010	0.038
SMG	-0.052 (-0.084, -0.020)	-0.021 (-0.052, 0.010)	0.169	0.298
STG	-0.087 (-0.119, -0.054)	-0.024 (-0.054, 0.007)	0.005	0.030
Mean thickness of cortical structure				
lOFC	-0.037 (-0.072, -0.001)	-0.005 (-0.039, 0.028)	0.198	0.299
SMG	-0.067 (-0.102, -0.033)	0.015 (-0.018, 0.047)	0.001	0.008
STG	-0.073 (-0.107, -0.039)	-0.008 (-0.040, 0.024)	0.006	0.030
Area of cortical structure				
lOFC	-0.064 (-0.096, -0.032)	-0.023 (-0.053, 0.007)	0.063	0.135
SMG	-0.028 (-0.060, 0.004)	-0.028 (-0.058, 0.003)	0.998	0.998
STG	-0.058 (-0.089, -0.026)	-0.034 (-0.063, -0.004)	0.275	0.360

Abbreviation: LGI, low-grade inflammation; CI, confidence intervals; FDR, false discovery rate; CN, caudate nucleus; PUT, putamen; THA, thalamus; lOFC, lateral orbitofrontal cortex; SMG, supramarginal gyrus; STG, superior temporal gyrus

a The reference is low-level LGI.

b The '*P* value FDR' is a measure of the error detection rate obtained by correcting for the difference significance P value. A low 'P value FDR value' (typically less than 0.05) suggests statistical significance.

Table S12. Associations of the brain structures with CRP and WBC across sex subgroups

Phenotype	CRP[a]				WBC[b]			
	β (95% CI)		*P* for interaction	*P* value FDR[c]	β (95% CI)		*P* for interaction	*P* value FDR[c]
	Male	Female			Male	Female		
Volume of subcortical structure								
CN	-0.026 (-0.061, 0.009)	-0.071 (-0.105, -0.037)	0.066	0.137	-0.061 (-0.095, -0.026)	-0.062 (-0.095, -0.029)	0.957	0.973
PUT	-0.026 (-0.059, 0.007)	-0.074 (-0.105, -0.042)	0.037	0.117	-0.081 (-0.113, -0.049)	-0.036 (-0.067, -0.006)	0.048	0.129
THA	-0.075 (-0.108, -0.042)	-0.098 (-0.129, -0.066)	0.326	0.399	-0.075 (-0.107, -0.043)	-0.019 (-0.050, 0.013)	0.013	0.043
Volume of cortical structure								
lOFC	-0.043 (-0.077, -0.010)	-0.073 (-0.105, -0.041)	0.204	0.299	-0.077 (-0.110, -0.044)	-0.032 (-0.063, 0.000)	0.048	0.129
SMG	-0.041 (-0.074, -0.007)	-0.055 (-0.087, -0.023)	0.553	0.620	-0.053 (-0.086, -0.020)	-0.016 (-0.048, 0.015)	0.118	0.215
STG	-0.058 (-0.092, -0.025)	-0.060 (-0.093, -0.028)	0.946	0.973	-0.085 (-0.118, -0.052)	-0.041 (-0.073, -0.009)	0.056	0.129
Mean thickness of cortical structure								
lOFC	-0.053 (-0.090, -0.017)	-0.025 (-0.060, 0.011)	0.260	0.360	-0.029 (-0.065, 0.007)	0.005 (-0.029, 0.040)	0.177	0.299
SMG	-0.067 (-0.103, -0.032)	-0.006 (-0.040, 0.029)	0.012	0.042	-0.015 (-0.050, 0.020)	0.029 (-0.004, 0.063)	0.072	0.144
STG	-0.080 (-0.116, -0.044)	-0.048 (-0.083, -0.014)	0.202	0.299	-0.041 (-0.076, -0.006)	0.007 (-0.027, 0.041)	0.051	0.129
Area of cortical structure								
lOFC	-0.019 (-0.052, 0.014)	-0.064 (-0.096, -0.033)	0.052	0.129	-0.064 (-0.096, -0.031)	-0.034 (-0.066, -0.003)	0.195	0.299
SMG	-0.017 (-0.050, 0.016)	-0.062 (-0.094, -0.030)	0.053	0.129	-0.050 (-0.082, -0.017)	-0.027 (-0.058, 0.005)	0.313	0.399
STG	-0.021 (-0.053, 0.012)	-0.058 (-0.090, -0.027)	0.100	0.194	-0.071 (-0.103, -0.039)	-0.056 (-0.086, -0.025)	0.499	0.599

Abbreviation: CRP, C-reactive protein; WBC, white blood cell (leukocyte) count; CI, confidence intervals; FDR, false discovery rate; CN, caudate nucleus; PUT, putamen; THA, thalamus; lOFC, lateral orbitofrontal cortex; SMG, supramarginal gyrus; STG, superior temporal gyrus

a The reference is low-level CRP.

b The reference is low-level WBC.

c The '*P* value FDR' is a measure of the error detection rate obtained by correcting for the difference significance P value. A low 'P value FDR value' (typically less than 0.05) suggests statistical significance.

Table S13. Associations of the brain structures with PLT and NLR across sex subgroups

Phenotype	PLT[a]				NLR[b]			
	β (95% CI)		*P* for interaction	*P* value FDR[c]	*β* (95% CI)		*P* for interaction	*P* value FDR
	Male	Female			Male	Female		
Volume of subcortical structure					-			
CN	0.022 (-0.013, 0.058)	-0.016 (-0.047, 0.016)	0.115	0.215	-0.014 (-0.046, 0.019)	0.017 (-0.016, 0.049)	0.202	0.299
PUT	-0.014 (-0.048, 0.019)	-0.010 (-0.039, 0.020)	0.840	0.916	-0.067 (-0.098, -0.037)	0.072 (0.041, 0.103)	<0.001	<0.001
THA	0.005 (-0.028, 0.039)	-0.038 (-0.067, -0.008)	0.060	0.133	-0.083 (-0.114, -0.052)	0.078 (0.047, 0.109)	<0.001	<0.001
Volume of cortical structure								
lOFC	0.011 (-0.023, 0.045)	-0.014 (-0.044, 0.015)	0.276	0.360	-0.058 (-0.089, -0.027)	0.056 (0.024, 0.087)	<0.001	<0.001
SMG	0.007 (-0.027, 0.041)	-0.019 (-0.049, 0.011)	0.264	0.360	-0.035 (-0.067, -0.004)	0.032 (0.001, 0.064)	0.003	0.002
STG	-0.006 (-0.040, 0.028)	-0.020 (-0.050, 0.010)	0.554	0.620	-0.058 (-0.089, -0.027)	0.046 (0.014, 0.077)	<0.001	<0.001
Mean thickness of cortical structure								
lOFC	0.033 (-0.004, 0.070)	-0.016 (-0.049, 0.017)	0.055	0.129	-0.029 (-0.063, 0.005)	0.04 (0.005, 0.074)	0.006	0.030
SMG	0.006 (-0.030, 0.042)	-0.026 (-0.058, 0.006)	0.191	0.299	-0.052 (-0.086, -0.019)	0.06 (0.027, 0.094)	<0.001	<0.001
STG	-0.003 (-0.039, 0.033)	-0.028 (-0.060, 0.004)	0.319	0.399	-0.033 (-0.066, 0.000)	0.073 (0.04, 0.107)	<0.001	<0.001
Area of cortical structure								
lOFC	-0.008 (-0.042, 0.026)	-0.005 (-0.035, 0.025)	0.894	0.941	-0.050 (-0.080, -0.019)	0.038 (0.007, 0.069)	<0.001	<0.001
SMG	0.004 (-0.030, 0.038)	-0.010 (-0.039, 0.020)	0.558	0.620	-0.019 (-0.050, 0.012)	0.009 (-0.022, 0.040)	0.210	0.300
STG	-0.008 (-0.041, 0.026)	-0.011 (-0.040, 0.018)	0.878	0.941	-0.051 (-0.081, -0.020)	0.010 (-0.021, 0.040)	0.007	0.032

Abbreviation: PLT, platelet count; NLR, neutrophil to lymphocyte ratio; CI, confidence intervals; FDR, false discovery rate; CN, caudate nucleus; PUT, putamen; THA, thalamus; lOFC, lateral orbitofrontal cortex; SMG, supramarginal gyrus; STG, superior temporal gyrus

a The reference is low-level PLT.

b The reference is low-level NLR.

c The '*P* value FDR' is a measure of the error detection rate obtained by correcting for the difference significance P value. A low 'P value FDR value' (typically less than 0.05) suggests statistical significance.

Table S14 Associations between the brain structures and low-grade inflammation-across genetic risk subgroups

Pheno-type	Low genetic risk [a]			Moderate genetic risk [a]			High genetic risk [a]			*P* for interaction
	β (95% CI)	*P* value	*P* value FDR[b]	*β* (95% CI)	*P* value	*P* value FDR	*β* (95% CI)	*P* value	*P* value FDR	
Volume of subcortical structure										
CN	-0.011 (-0.051, 0.029)	0.587	0.774	-0.052 (-0.092, -0.012)	0.010	0.034	-0.059 (-0.099, -0.019)	0.004	0.024	0.194
PUT	-0.050 (-0.088, -0.013)	0.009	0.033	-0.057 (-0.094, -0.020)	0.003	0.021	-0.035 (-0.072, 0.002)	0.066	0.140	0.700
THA	-0.050 (-0.088, -0.012)	0.009	0.033	-0.062 (-0.100, -0.025)	0.001	0.021	-0.060 (-0.098, -0.022)	0.002	0.021	0.889
Volume of cortical structure										
lOFC	-0.041 (-0.079, -0.003)	0.034	0.096	-0.051 (-0.089, -0.013)	0.009	0.033	-0.058 (-0.096, -0.020)	0.003	0.021	0.826
SMG	-0.025 (-0.063, 0.014)	0.204	0.306	-0.032 (-0.070, 0.007)	0.106	0.196	-0.051 (-0.089, -0.013)	0.009	0.033	0.613
STG	-0.036 (-0.074, 0.003)	0.067	0.140	-0.058 (-0.096, -0.020)	0.003	0.021	-0.067 (-0.105, -0.029)	0.001	0.021	0.501
Mean thickness of cortical structure										
lOFC	-0.022 (-0.064, 0.020)	0.296	0.418	-0.031 (-0.073, 0.011)	0.143	0.245	-0.006 (-0.048, 0.036)	0.774	0.826	0.696
SMG	-0.011 (-0.052, 0.030)	0.598	0.774	-0.034 (-0.074, 0.007)	0.101	0.194	-0.026 (-0.067, 0.014)	0.202	0.306	0.724
STG	-0.036 (-0.077, 0.004)	0.080	0.160	-0.047 (-0.088, -0.007)	0.023	0.074	-0.033 (-0.073, 0.008)	0.115	0.204	0.876
Area of cortical structure										
lOFC	-0.037 (-0.074, 0.001)	0.058	0.139	-0.037 (-0.075, 0.000)	0.052	0.131	-0.054 (-0.092, -0.017)	0.005	0.027	0.757
SMG	-0.025 (-0.063, 0.013)	0.201	0.306	-0.020 (-0.058, 0.017)	0.291	0.418	-0.039 (-0.077, -0.001)	0.042	0.112	0.768
STG	-0.036 (-0.073, 0.002)	0.062	0.140	-0.042 (-0.079, -0.004)	0.028	0.084	-0.058 (-0.096, -0.021)	0.002	0.021	0.681

Abbreviation: LGI, low-grade inflammation; CI, confidence intervals; FDR, false discovery rate; CN, caudate nucleus; PUT, putamen; THA, thalamus; lOFC, lateral orbitofrontal cortex; SMG, supramarginal gyrus; STG, superior temporal gyrus

a The reference is low-level LGI;

b The '*P* value FDR' is a measure of the error detection rate obtained by correcting for the difference significance P value. A low 'P value FDR value' (typically less than 0.05) suggests statistical significance.
